# Changes in cAMP effector predominance are associated with increased oxytocin receptor expression in twin but not infection-associated or idiopathic preterm labour

**DOI:** 10.1371/journal.pone.0240325

**Published:** 2020-11-30

**Authors:** Angela Yulia, Alice J. Varley, Natasha Singh, Kaiyu Lei, Rachel Tribe, Mark R. Johnson

**Affiliations:** 1 Institute of Reproductive and Developmental Biology, Imperial College School of Medicine, Chelsea and Westminster Hospital, London, United Kingdom; 2 Department of Women and Children’s Health, School of Life Course Sciences, Kings College London, London, United Kingdom; John Hunter Hospital, AUSTRALIA

## Abstract

We previously reported that at term pregnancy, a decline in myometrial protein kinase A (PKA) activity leads to an exchange protein activated by cyclic AMP (Epac1)-dependent increase in oxytocin receptor (OTR) expression, promoting the onset of labour. Here, we studied the changes in the cyclic adenosine monophosphate (cAMP) effector system present in different phenotypes of preterm labour (PTL). Myometrial biopsies obtained from women with phenotypically distinct forms of PTL and the levels of PKA and OTR were examined.

Although we found similar changes in the cAMP effector pathway in all forms of PTL, only in the case of twin PTL (T-PTL) was myometrial OTR levels increased in association with these results. Although there were several changes in the mRNA levels of components of the cAMP synthetic pathway, the total myometrial cAMP levels did not change with the onset of any subtype of PTL. With regards to the expression of cAMP-responsive genes, we found that the mRNA levels of 4 of the 5 cAMP-down-regulated genes were increased in T-PTL, similar to our findings in term labour.

These data signify that although changes in the cAMP effector system were common to all forms of PTL, only in T-PTL were OTR levels increased. Similarly, the mRNA levels of cAMP-repressed genes were only increased in T-PTL supporting the concept that the decline in PKA levels influences myometrial function driving the onset of T-PTL.

## Introduction

Understanding the factors that control the onset of labour is key to our ability to stop and start labour when medically necessary. Preterm labour (PTL) remains the most important cause of childhood mortality globally [1] and current approaches to its management are relatively unsuccessful [2]. Consequently, understanding the processes that underpin normal and abnormal labour is critical if we are to reduce the impact of preterm birth.

Cyclic AMP (cAMP) is widely expressed and acts as a second messenger in intracellular signalling pathways controlling a wide range of cellular functions. In the myometrium, it acts via its primary effector, protein kinase A (PKA), to promote uterine quiescence. cAMP main effector, PKA, is regulated by transcription factors [3,4]. The regulation of transcription by PKA is mainly achieved by direct phosphorylation of the transcription factors cAMP-response element-binding protein (CREB), and cAMP-responsive modulator (CREM) [3,4]. The phosphorylation process is essential because it allows these proteins to interact with the transcriptional coactivators CREB-binding protein (CBP) [3]. The CREM gene also encodes the powerful repressor ICER, which negatively feeds back on cAMP-induced transcription [3,5]. These transcription factors can all be phosphorylated by many different kinases, for example, CREB is activated by phosphorylation at Ser133 by various signalling pathways including extracellular signal-regulated kinase, and calcium [5,6]. Our group have previously demonstrated that the decline in PKA activity with advancing gestation was supported by demonstrating a reduction in phospho-CREB, with the lowest levels observed at the term established labour group [7].

Our recent paper described how a decline in myometrial PKA activity at term was associated with an increase in the key pro-labour factor, the oxytocin receptor (OTR) [7]. In our previous study, we performed a gene array on myometrial cells exposed to forskolin 100 μM for 6 hours and used a gene array to identify the cAMP-responsive genes; five genes were down-regulated by forskolin, *PRKG1*, *GUCY1A3*, *GPR124*, *CREB3L1*, and *OTR*, and six genes were up-regulated by forskolin, *MKP-1*, *11*β*HSD1*, *PTGES*, *PDE4B*, *GPR125*, and *CCL8* [7]. As well as forskolin, we confirmed the ability of other cAMP agonists, 8-bromoadenosine-cAMP, dibutyryl-cAMP and rolipram, to reproduce the changes in gene expression observed in the gene array experiment. We found previously, that with the onset of labour, the repressed genes behaved most consistently, with four out of five, increasing with advancing gestation and the onset of labour. Importantly, in the case of OTR, a pro-labour gene, the changes in mRNA were accompanied by a similar change in protein levels and response to oxytocin [7].

We observed that cAMP levels were highest in myometrial samples from the term no labour group but declined in early labour myometrial samples to the levels observed in preterm no labour samples and remained at this level in established labour samples. In the same samples, OTR mRNA and protein expression increased with gestation and the onset of labour, reaching a peak in term early labour, before declining in term established labour. PKA regulatory subunit IIα (PKAR2α) levels (mRNA and protein) were highest in the preterm no labour samples and declined progressively thereafter. Epac1 mRNA levels increased progressively from preterm no labour to term established labour, peaking in the term established labour myometrial samples, but the protein levels peaked in the term early labour samples [7]. Our *in vitro* studies showed that reducing PKA resulted in an increase in OTR expression in an Epac1-dependent manner, suggesting that the decline in myometrial PKA and increase in myometrial Epac1 may be responsible for the labour-associated increase in OTR which is critical for the onset of human labour [7]. Previously, others have demonstrated that the cAMP maintenance of myometrial quiescence is associated with an increase in the levels of the GTP-binding protein (Gαs). In contrast we found that although GαS mRNA expression was highest in the term early labour samples and reduced in the term established labour samples, the protein levels were unchanged across gestation and with labour [7]. Collectively, the data suggest that the well-recognised reduction in myometrial PKA activity [7–9] is associated with an increase in OTR gene expression, protein levels and the functional response to oxytocin. These data are highly significant for our understanding of the onset of term labour. The key question now is; are they reproduced in PTL? If they are, then this offers the prospect of potential therapeutic targets upstream of the OTR, the inhibition of which is only moderately effective in arresting PTL once it has started [10].

In this paper, we describe the changes in the cAMP effector system present in different phenotypes of PTL to test the hypotheses that these variations in effector predominance are common to all forms of PTL and associated with an increase in OTR levels. These results will help us to design therapeutic interventions for specific forms of PTL.

## Materials and methods

### Tissue specimens

Myometrial biopsies (0.5 x 0.5 x 0.5 cm^3^) were taken from the upper margin of the lower segment uterine incision at the time of women undergoing Caesarean section at Chelsea and Westminster Hospital. For the analysis of changes in PTL, all samples included in the study were obtained from women who were either non-labouring or labouring between 24 and 36 weeks gestation. Following collection, tissues were immediately snap frozen at -80°C for subsequent mRNA and protein extraction.

All patients were fully informed prior to the procedure and provided written consent for inclusion in the research study. Approval from The London-Chelsea Ethics committee for myometrial biopsy collection was obtained.

### PTL samples

These were classified into phenotypically defined groups; emergency Caesarean sections were performed for breech presentation or fetal distress in early labour, defined by a cervical dilatation of less than 3 cms [11]. The demographic details are summarised in S1 Table. The groups were as follows:

- *Preterm not in labour (PTNL)–*elective Caesarean section indicated for pre-eclampsia or fetal growth restriction.
- *PTL as a result of chorioamnionitis (CA-PTL)–*preterm delivery by emergency Caesarean section in the context of clinical findings include fever, uterine fundal tenderness, maternal or fetal tachycardia and purulent offensive vaginal discharge, which is associated with maternal leukocytosis and/or bacteraemia.
- *Idiopathic PTL (I-PTL)–*preterm delivery by emergency Caesarean section due to spontaneous PTL with no associated evidence of infection, abruption, polyhydramnios and excluding fibroids, previous cervical surgery, mid-trimester loss, previous pelvic inflammatory disease or sexually transmitted infections.
- *Preterm twin labour (T-PTL)/preterm twin no labour (T-PTNL)–*uncomplicated twin pregnancies delivered by elective Caesarean section or after the spontaneous of PTL by emergency Caesarean section with no evidence of infection, abruption or uterine anomaly.

## Methods

### Protein extraction

Protein was extracted from ‘pre-prepared’ tissue samples: the myometrial tissue samples were cut and weighed to predetermined amounts on dry ice and then place in precooled precellys tubes prior to the homogenisation steps. The myometrial tissue samples were homogenised in lysis buffer using the Precellys®24 Dual system. The samples were placed in Precellys tube type (CK Mix #03961-1-00) and spun at 5000 rpm for 20 seconds. The homogenate was centrifuged for 15 minutes at 5000 rpm and the total protein was extracted. The concentrations of the protein were determined by protein assay (Bio-Rad Laboratories, USA) using bovine serum albumin (BSA) as reference standards. The protein concentration of standards and samples were measured using a spectrophotometer at a wavelength of 660nm. A standard curve was calculated from the absorbance of the standards and used to determine the sample protein concentration.

### Western blotting

Protein samples were denatured following the addition of NuPAGE LDS loading buffer (Invitrogen, UK) and heated at 70°C for 10 minutes. 20μg protein samples were loaded into each well on polyacrylamide (Tris-glycine) gels alongside the Precision Plus Protein™ pre-stained standards (Bio-Rad, Hertfordshire, UK). The gels were subjected to SDS-PAGE electrophoresis and subsequently transferred onto polyvinylidene fluoride (PVDF) membranes using the Trans-Blot^®^ Turbo^TM^ Transfer System (Bio-Rad, Hertfordshire, UK). The membranes were blocked with 5% w/v fat-free milk in Tween-Tris-buffered saline (TBS-T) solution for 1 hour at room temperature and then hybridized with the primary antibody overnight at 4°C [S2 Table]. The membranes were washed for 1 hour prior to incubation with the secondary antibody at a 1:2000 dilution for 2 hours at room temperature. ECL plus (GE Healthcare, Little Chalfont, UK) was used for antibody detection. A representative western blot is shown next to each graph displaying the densitometry of the protein levels.

### RNA extraction

Total RNA was extracted from pre-prepared primary myometrial tissue samples and purified using TRIzol Plus RNA Purification kit (Life Technologies, Dartford, UK) to measure mRNA levels.

### Quantitative RT-PCR

Following quantification, 1.5μg of RNA was reverse transcribed with oligo DT random primers and MuLV reverse transcriptase using the QuantiTect Reverse Transcription kit (Qiagen, Manchester, UK). Primer sets [S3 Table] were designed and purchased from Invitrogen (Paisley, UK). Quantitative PCR was performed in the presence of SYBR Green (Roche, West Sussex, UK) using RotorGene Q thermocycler (Qiagen, Manchester, UK). The pre-PCR cycle occurred for 10 minutes at 95°C followed by up to 45 cycles at 95°C for 20 seconds, 58–60°C for 20 seconds and 72°C for 20 seconds, with an extension at 72°C for 15 seconds. The final process involves a melt over a temperature range of 72–99°C rising by 1°C steps, followed by a wait of 15 seconds on the first step and a wait of 5 seconds for each subsequent step. The cycle threshold (Ct) was used for quantitative analysis, which denotes the cycle at which fluorescence reaches a preset threshold. The cycle threshold is set at a level whereby the exponential increase in amplicon yield is approximately parallel between the samples. All mRNA abundance data were expressed relative to the amount of the constitutively expressed GAPDH. We previously have trialled and used GAPDH as housekeeping gene as we identified that it was the best reference gene in myometrial samples [11].

### Cyclic AMP assay on myometrial tissue

Intracellular cAMP was measured using the cAMP chemiluminescent immunoassay kit (Ann Arbor Assays) as previously described [7]. Frozen preterm myometrial tissue biopsies were grinded in a stainless steel mortar under liquid nitrogen until they were in a fine powder form. The powdered tissues were weighed and 1ml of sample diluent was added for every 100mg of tissue. The tissues were then incubated in the sample diluent for 10 minutes on ice, and were then centrifuged at≥ 600x g at 4°C for 15 minutes. The supernatants were collected and assays were performed using High Sensitivity Direct Cyclic AMP Chemiluminescent Immunoassay Kit (Arbor Assay, USA) according to the manufacturer’s protocol. The binding reaction is initiated by the addition of a polyclonal antibody to cAMP. After a 2 hour incubation the plate is washed and chemiluminescent substrate is added. The substrate immediately reacts with the bound cAMP-peroxidase conjugate. The generated chemiluminescent glow signal is measured. The concentration of the cAMP in the sample is calculated, after making correction for the dilution. These are the n values for the levels of cAMP; PTNL n = 19, CA-PTL n = 14, I-PTL n = 7, T-PTNL n = 13, T-PTL n = 6.

### Statistical analysis

All data were initially tested for normality using a Kolmogorov–Smirnoff test or a D’Agostino & Pearson normality test depending on the group size. Normally distributed data were analysed using a Student’s t-test for two groups or an ANOVA followed by a Dunnett’s or Bonferroni’s post-hoc test for three groups or more. Data which were not normally distributed were analysed using a Wilcoxon matched pair or a Mann-Whitney test for unpaired data for 2 groups or a Friedman’s test with a Dunn’s multiple comparisons post-hoc test to compare three groups or more. P <0.05 was considered statistically significant. The GraphPad Prism 7.0 software was used to generate graphical representations of the data.

## Results

### Changes in myometrial cAMP effector system in PTL

**Fig 1:**
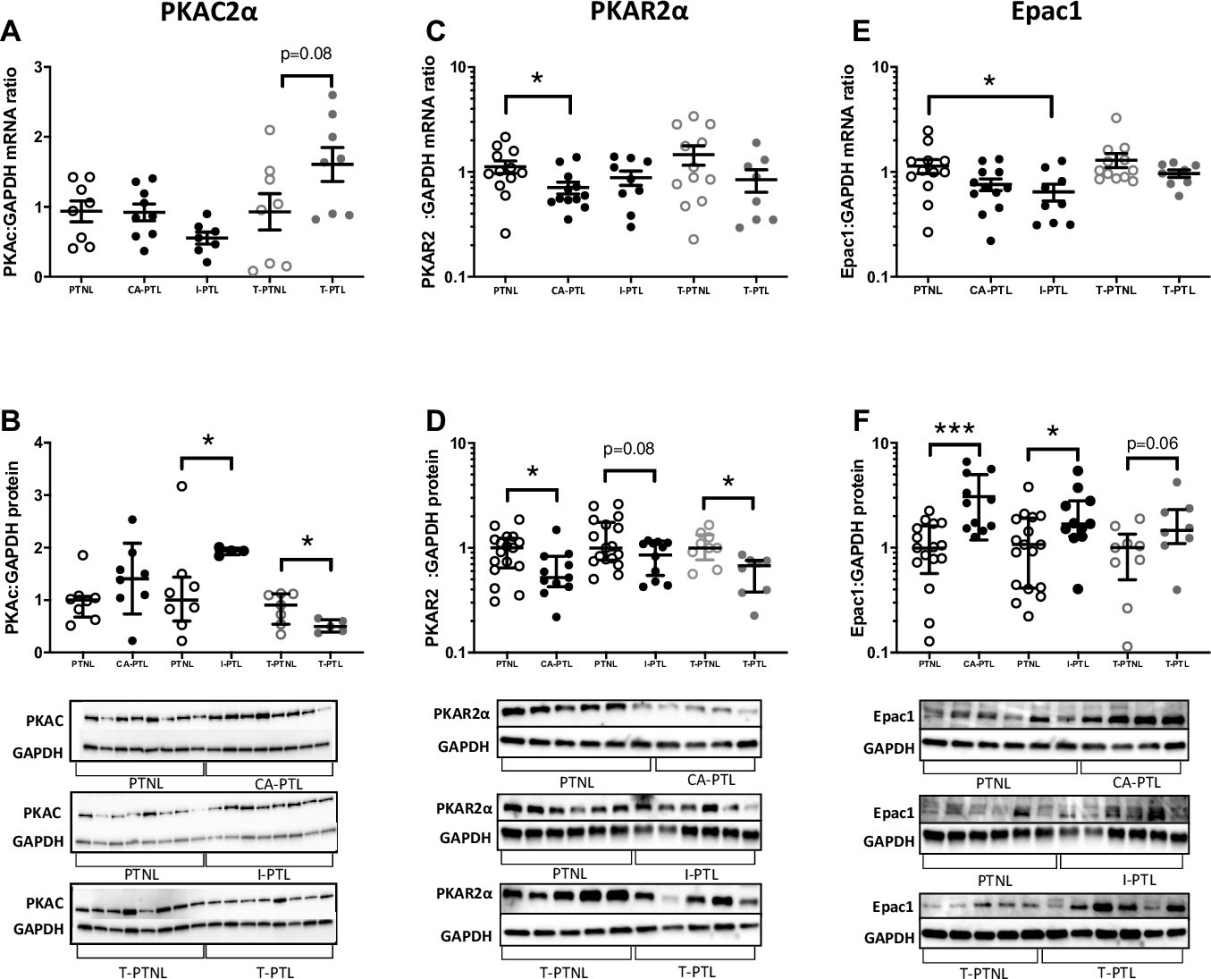
Myometrial cAMP-effector levels in preterm labour myometrial samples. Human myometrial tissue samples were snap frozen at -80°C for mRNA and protein extraction. The levels of PKAc, PKAR2α, Epac1 mRNA **(A, C, E)** and protein **(B, D, F)** were measured using quantitative rt-PCR and western blotting respectively. Blots were probed with PKAc, PKAR2α, and Epac1 antibody, and GAPDH was used as a loading control. These are the n values for protein samples; PKAc: PTNL (chorio) n = 8, CA-PTL n = 8, PTNL (idio) n = 8, I-PTL n = 4, T-PTNL n = 7, T-PTL n = 5; PKAR2α and Epac1: PTNL (chorio) n = 17, CA-PTL n = 11, PTNL (idio) n = 17, I-PTL n = 11, T-PTNL n = 9, T-PTL n = 8. *P<0.05, **P<0.01, ***P<0.001 (n = 4–17 in each group).

In T-PTL samples, although there is no significant difference in mRNA levels (Fig 1A, 1C & 1E), both PKAc and PKAR2α declined at a protein level (both p<0.05; Fig 1B & 1D) and Epac1 protein level tended to increase (p = 0.06; Fig 1F). In I-PTL samples, there is no significant difference in PKAc and PKAR2α mRNA levels (Fig 1A & 1C), the Epac1 mRNA level was however significantly reduced (p<0.05; Fig 1E). In I-PTL protein samples, the PKAc levels increased (p<0.05; Fig 1B), PKAR2α tended to decline (p = 0.08; Fig 1D) and Epac1 levels increased (p<0.05; Fig 1F). Finally, in CA-PTL samples, PKAR2α declined (p<0.05; Fig 1C & 1D) and Epac1 protein levels increased (p = 0.001; Fig 1F). There is no significant difference in mRNA and protein levels of PKAc and Epac1 mRNA levels (Fig 1A, 1B & 1E).

### Myometrial cAMP synthesis in PTL

**Fig 2:**
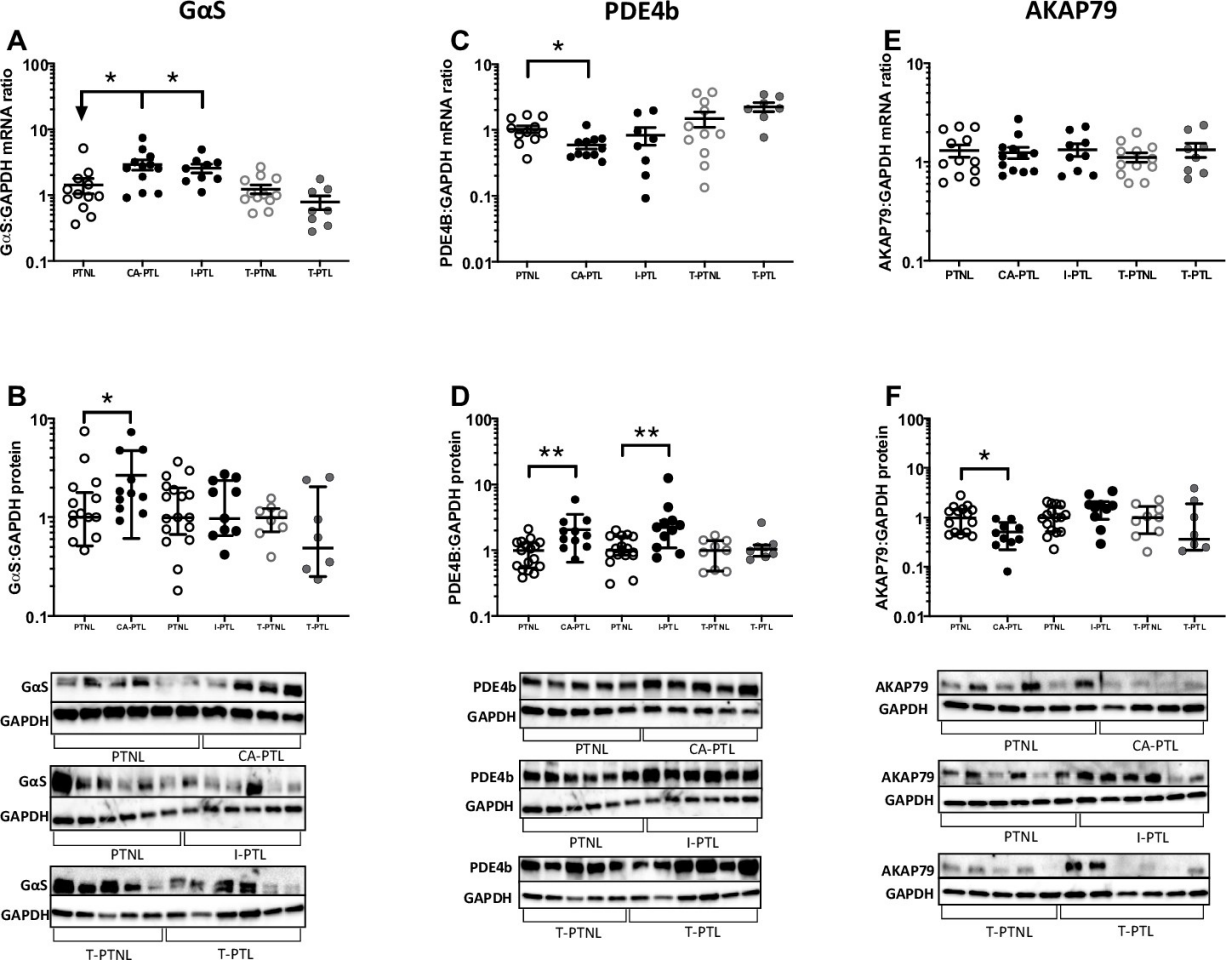
The levels of G-protein alpha subunit (GαS), PDE4B, and AKAP79 in preterm myometrium. Human myometrial tissue samples were snap frozen at -80°C for mRNA and protein extraction and cAMP analysis. The levels of GαS, PDE4B, and AKAP79 mRNA **(A, C, E)** and protein **(B, D, F)** were measured using quantitative rt-PCR and western blotting respectively. Blots were probed with GαS, PDE4B, and AKAP79 antibody, and GAPDH was used as a loading control. These are the n values for protein samples; GaS: PTNL (chorio) n = 17, CA-PTL n = 11, PTNL (idio) n = 17, I-PTL n = 11, T-PTNL n = 8, T-PTL n = 8; PDE4B PTNL (chorio) n = 17, CA-PTL n = 11, PTNL (idio) n = 17, I-PTL n = 11, T-PTNL n = 9, T-PTL n = 8; AKAP79: PTNL (chorio) n = 16, CA-PTL n = 11, PTNL (idio) n = 16, I-PTL n = 10, T-PTNL n = 9, T-PTL n = 8. *P<0.05, **P<0.01, ***P<0.001 (n = 6–17 in each group).

Others have reported changes in GαS, PDE4b and AKAP79 with term labour [8,9,12]. Here we found that in protein samples, GαS increased in CA-PTL (p<0.05; Fig 2B), PDE4b increased in CA-PTL and I-PTL (both p<0.01; Fig 2D) and AKAP79 was reduced in CA-PTL (p<0.05; Fig 2F). In mRNA samples, GαS increased in CA-PTL and I-PTL (both p<0.05; Fig 2A), PDE4b decreased in CA-PTL (p<0.05; Fig 2C), and there is no significant difference in AKAP79 in all phenotype of preterm labour (Fig 2E). Of the adenylyl cyclases (AC) protein samples, only AC2 protein levels in I-PTL was increased (p<0.01; S1B Fig). There is a significant reduction in AC2 mRNA samples in CA-PTL and I-PTL (p<0.01 and p<0.05; S2A Fig). There is significant increase in AC3 mRNA samples in T-PTL (p<0.05; S2C Fig), and significant reduction in AC9 mRNA samples in CA-PTL and I-PTL (p<0.01 and p<0.05; S2E Fig). Consistent with the lack of coherent change in the cAMP synthetic/metabolic components, myometrial cAMP levels did not change across different types of PTL (S2 Fig).

**Fig 3:**
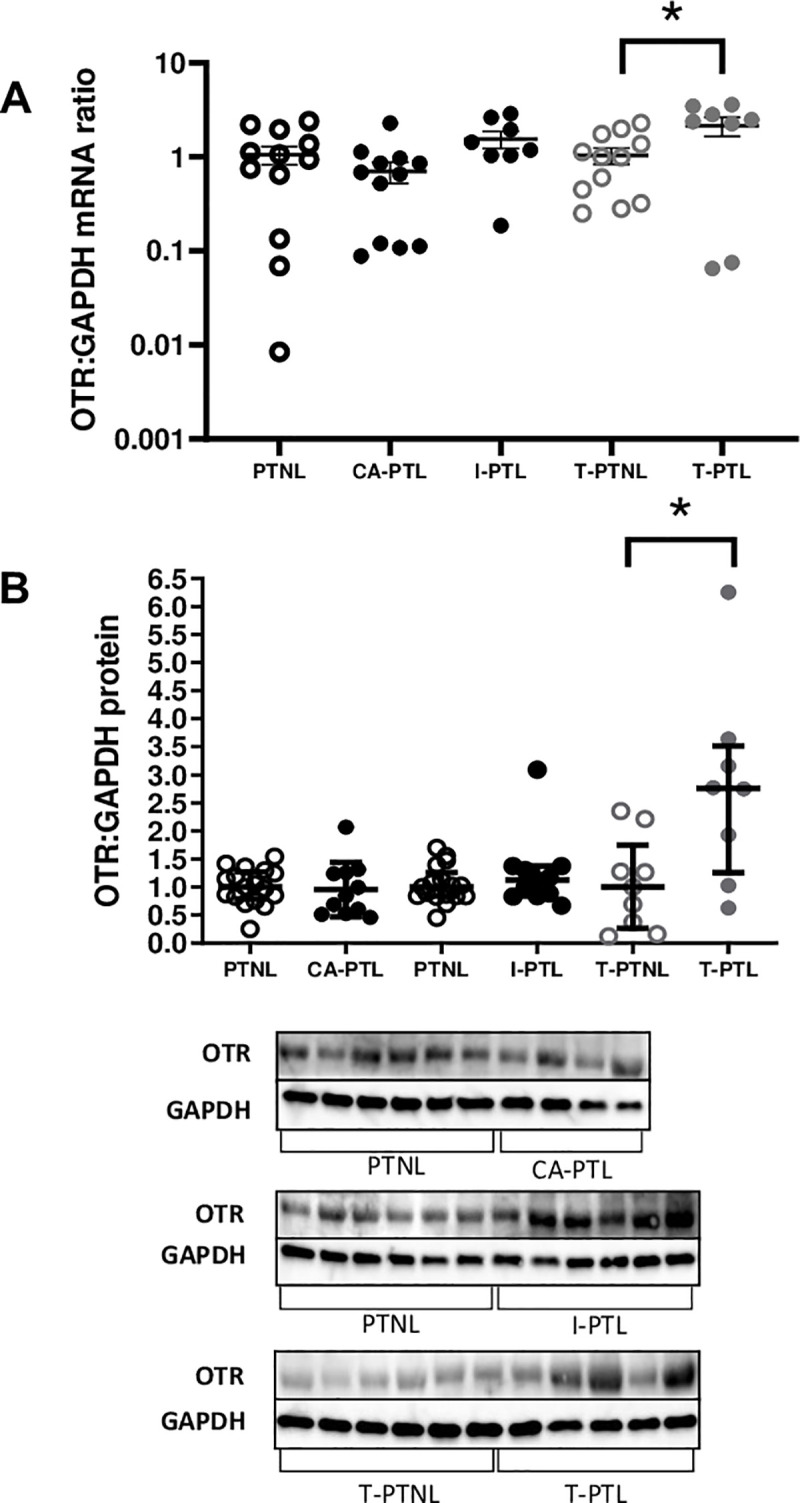
Myometrial OTR levels in preterm labour. Human myometrial tissue samples were snap frozen at -80°C for mRNA and protein extraction. **A.** The levels of OTR mRNA **(A)** and protein **(B)** were measured using quantitative rt-PCR and western blotting respectively. Blots were probed with OTR antibody, and GAPDH was used as a loading control. The n no for OTR protein: PTNL (chorio) n = 17,CA-PTL n = 11, PTNL (idio) n = 17, I-PTL n = 11, T-PTNL n = 9, T-PTL n = 7*P<0.05, **P<0.01, ***P<0.001 (n = 6–17 in each group).

**Fig 4:**
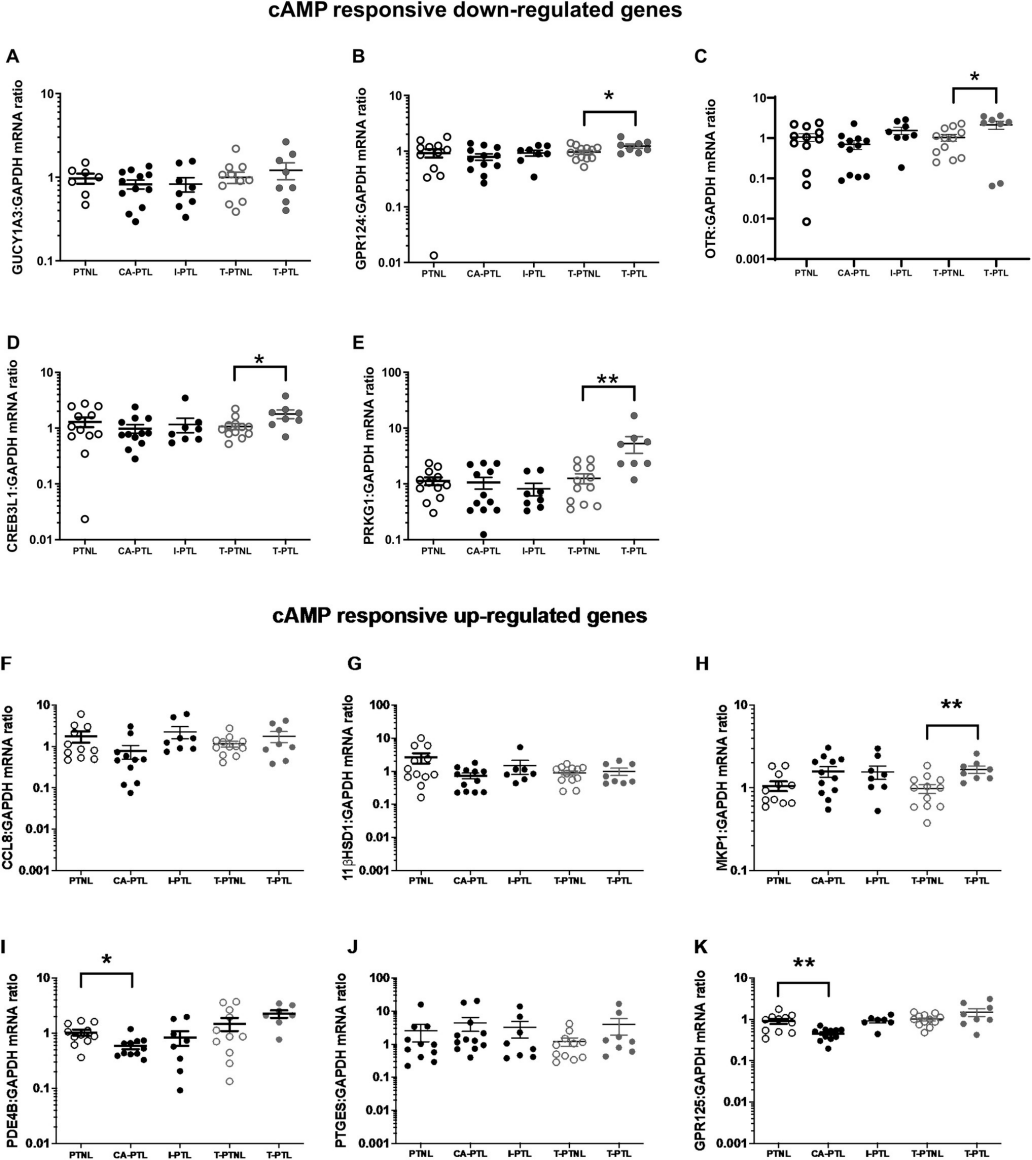
The levels of cAMP regulated gene expression in preterm myometrium. Human myometrial tissue samples were snap frozen at -80°C for mRNA extraction. The mRNA levels of the “down-regulated” genes GUCY1A3, OTR, GPR124, CREB3L1 and PRKG1 mRNA (A-E) and of the “up-regulated” genes CCL8, 11βHSD1, MKP1, PDE4B, PTGES and GPR125 mRNA (F-K) were measured using quantitative rt-PCR. *P<0.05, **P<0.01, ***P<0.001 (n = 6–14 in each group).

In the current study, OTR mRNA (p<0.05; Fig 3A) protein levels (p<0.05; Fig 3B) were increased in T-PTL myometrial samples only. We examined the expression of other cAMP-responsive genes and found that similar to our observation at term [7], 4 of the 5 cAMP down-regulated genes were increased in T-PTL (p<0.05–0.01; Fig 4B–4E). Neither CA-PTL or I-PTL influenced the expression of cAMP down-regulated genes (Fig 4A–4E). Again, similar to our observations in term tissues, the expression of cAMP up-regulated genes was inconsistent, with 2 of the 6 up-regulated genes reduced in CA-PTL (p<0.05 and 0.01; Fig 4I & 4K) and 1 gene increased in T-PTL (p<0.01; Fig 4H).

### Myometrial cAMP-related transcription factors in PTL

Protein levels of CBP were reduced in CA-PTL and increased in I-PTL (both p<0.001; S3B Fig), protein levels of ICER were increased in CA-PTL (p<0.01; S3D Fig) and CREB protein levels were reduced in both CA-PTL and T-PTL (p<0.05 and 0.001 respectively; S3F Fig), but phospho-CREB levels were unaltered (S3G Fig).

There are some discrepancy between the mRNA and protein levels in our results above. This is because we studied one time point rather than a series of points, meaning that we could not follow the increase in transcription (mRNA levels) and the subsequent increase in translation (protein levels). The use one time point and/or differences in mRNA and protein half lives could both result in the observed discrepancy. The process is further complicated by changes in MAPK activity, which is known to be altered in labouring myometrium, have been shown to change the efficiency of translation, such that protein levels may rise in the absence of any change in transcription (mRNA levels). Similarly, increased miRNA levels might inhibit translation meaning that increased mRNA levels might not be relflected in changes in the related protein levels. We agree that measuring mRNA is a good indicator of gene expression, but in our studies to date we do not always find a total agreement between mRNA levels and the associated protein levels, we believe that there are too many variables for this to be uniformly the case.

## Discussion

We observed a decline in the cAMP/PKA system in T-PTL in association with an increase in OTR levels and a loss of repression of cAMP repressed genes, as we reported in early term labour [7]. Although there were similar changes in the cAMP/PKA system in other forms of PTL, they were not associated with increases in OTR expression or the mRNA levels of other cAMP/PKA repressed genes suggesting that these changes may not be significant in the onset of CA- or I-PTL. These data imply that the changes in the cAMP effector system are influential in the final common pathway leading to twin PTL and taken with our data at term [7]. They suggest that the cAMP effector system is a potential therapeutic target in the modulation of labour.

### Cyclic AMP

In our previous work, we found that myometrial cAMP levels were highest in the term no labour group but declined in early labour to the preterm no labour levels and remained at this level in established labour [7]. In the current study, the lack of change from the preterm no labour samples compared to the labouring samples is consistent with our original observation, ie that early labour samples had similar levels to the preterm no labour samples. We did discover that AKAP79 levels were reduced in CA-PTL samples and that PDE4B levels were increased in both CA-PTL and I-PTL samples. As the exact mechanism through which cAMP induces a specific cellular response to promote myometrial relaxation remains uncertain, these results imply that the subcellular localisation of cAMP activation may change with the onset of CA-PTL and I-PTL. Clearly, such findings were not associated with altered patterns of gene expression, but they could alter myometrial contractility directly by other mechanisms. This would be consistent with the compartmentalisation of cAMP signalling in other tissues [13], which is facilitated by scaffold proteins. Scaffold proteins bring specific components of the signalling pathway together to ensure the precise subcellular targeting of cAMP actions [14,15]. Plasma membrane AKAPs, have high affinity, and thus bind with RII subunits of PKA to form AKAP–RII complexes. Studies on the functional effects the AKAP–RII complexes suggest that PKA anchoring may enhance the interactions of PKA with its physiological substrates and, in pregnant human myometrium, that this may be fundamental in maintaining uterine quiescence [15]. For example, with regards to AKAP79, PKAR2α formed complexes with AKAP79, and study has shown that PKAR2α localized to the plasma membrane via binding to AKAP79 was necessary for the cAMP inhibitory effect on myometrial phosphatidylinositide/calcium signalling pathways during gestation, thus the PKAR2α-AKAP29 complexes contributes to myometrial quiescence [16]. The lower level of AKAP79 in CA-PTL results in less PKAR2α-AKAP29 complexes, thus lowering the cAMP’s myometrial quiescence and eventually contraction starts. Moreover, this increase in RIIα protein also gives rise to increased particulate type II PKA catalytic activity in membranes prepared from term nonlaboring myometrium in comparison with labouring and non-pregnant membrane preparations. These data indicated that increased particulate type II PKA activity occurs during gestation, which would appear to be involved in directing the cAMP quiescence signal to specific subcellular loci within myometrial cells including the contractile machinery at the cytoskeleton; this effect is then removed during parturition. These data also suggested that PKA localized to the plasma membrane via binding to AKAPs was necessary for the cAMP inhibitory effect on myometrial phosphatidylinositide/calcium signalling pathways during gestation. Consequently the possibility exists that a similar mechanism occurs in the human myometrium during gestation and labour.

Tissue concentration may not reflect any change in sub-cellular micro-domain concentrations of cAMP. Subsequently, although we found no change in myometrial cAMP total levels, that does not exclude changes in subcellular microdomain cAMP concentrations. Bailey et al., studied the patterns of expression of various isoforms of these proteins in the human uterus using electrophoretic mobility shift and super shift assays, as well as immunoblotting of paired myometrial tissue samples from non-pregnant, pregnant non-labouring and spontaneous labouring women [17]. They reported spatio-temporal changes in the expression of cAMP associated gene expression, which are differentially expressed, depending on the gestational state of the uterus, all of which may have an important role in the control of myometrial quiescence [17].

With regards to the PDE4 family, they which specifically hydrolyses cAMP, and they are predominantly expressed in inflammatory cells and in smooth muscles of the myometrium. The levels of PDE4B are markedly abundant in the myometrium. Thus, increased levels of PDE4B will metabolise PKA, lowering its level and its ability to maintain uterine quiescence, and contraction begins [18]. Certainly, inhibition of PDE activity promotes myometrial relaxation *in vitro* [18] and *in vivo* inhibits LPS-induced parturition in the mouse [19]. In addition, PDEs play a critical role in the maintenance of microdomain cAMP levels [18]. At the whole tissue level, we have shown that increasing cAMP levels promotes relaxation of human myometrial strips [20], but the exact mechanism remains unclear. We have also shown that cAMP enhances progesterone action *in vitro* [21] and our latest data in the mouse suggests that this is also true *in vivo* [22], raising the possibility that this interaction is important in the regulation of myometrial contractility.

### Early labour

We selected cases of PTL who were in early labour only; this is defined by painful contractions and a cervical dilatation of less than 3 cms. This definition is clinically based and is a potential weakness in case selection as it is based on an individual’s perception of pain and uses the cervix as an indicator of effective myometrial contractions. What we are trying to identify is the stage of labour where the factors responsible for the initiation of myometrial contractions may still be apparent, before they are obscured by the secondary changes of labour itself. The significance of this is highlighted by our earlier work that showed that myometrial inflammation is a consequence of term labour and not a cause [11]. Others have reported that OTR expression reduces with labour duration [23]. Together, these reports clearly illustrate the importance of correctly identifying the stage of labour. The weakness of our current definition is that it assumes that every cervix has a similar compliance and will dilate at a constant rate in response to the same strength of contractions, when clearly this is not the case. Finding a better way to define the stage of labour is crucial if we are to understand the factors that control the onset of labour.

As changes in the cAMP effector system are present in all forms of PTL, in terms of either a reduction in PKAR2α or Epac1, this implies that targeting this system may be a viable therapeutic approach in the prevention or treatment of PTL. Equally, when the cAMP system was targeted indirectly previously, using beta-mimetics in women with threatened PTL, the efficacy of these agents was variable, particularly with longer-term administration, and they were associated with severe side effects [24]. Their use in the UK at least has largely been abandoned, with the exception of acute administration in cases of uterine hyperstimulation [25]. The loss of efficacy with time was thought to be due to receptor desensitization [26], however our data offer an alternative explanation. Previously, we showed in primary myometrial cells, that as PKA activity declined cAMP signals through an alternative cAMP effector system, Epac1, to drive OTR expression [7]. The changes we observed in all forms of PTL suggest that the changes in intracellular cAMP levels might activate Epac1 and increase OTR expression, which may potentially result in an increase in myometrial contractility. These data provide an alternative explanation for the loss/lack of consistent effect of β2 adrenergic receptor agonists in the management of PTL.

GαS levels were increased in CA-PTL and AC2 protein levels were increased in I-PTL only, but since cAMP levels were unchanged, the significance of these findings is doubtful. The GαS data are consistent with our term data [7], but contrast with other reports of a decline in levels in term labouring myometrial samples [9]. There are little data regarding myometrial AC levels, one previous publication showed that AC I, II, III, VIII and IX were present in human myometrium, but did not compare changes with labour onset [27]. In terms of cAMP metabolism, we studied phosphodiesterase (PDE) type 4b levels as these have been shown to be the predominant PDE isoform in pregnant myometrium [28] and found that the protein levels of both were increased in both CA-PTL and I-PTL. PDEs metabolise cAMP, converting it to AMP and terminating its action, thus higher levels should reduce cAMP levels, but we found no evidence of a reduction in total cAMP levels in this study. Equally, PDE’s often act to regulate cAMP concentrations in defined subcellular regions, which we would not detect measuring total tissue content of cAMP.

In summary, although myometrial cAMP levels was not different in each of the phenotypes of PTL examined in this study, we observed a decline in PKAR2α and a trend for Epac1 levels to increase. The reduction in AKAP79 in CA-PTL, the increase CBP in I-PTL, and the reduction of CREB protein levels in both CA-PTL and T-PTL shown in this study support the concept that that stretch and inflammation are important in the changes in cAMP effector system, all of which may be crucial in contributing to the final common pathway of PTL. Indeed, we have recently shown that stretch and inflammation alone increased OTR protein levels; however, the increase in OTR levels induced by the combination of stretch and inflammation was not greater than either stimulus alone [7].

The role for stretch in the expression of pro-labour proteins has been investigated extensively in our group; we have previously shown that stretch of human myometrial cells *in vitro* results in up-regulation of cyclooxygenase-2 (COX-2) mRNA expression and protein synthesis in association with activator protein-1 (AP-1) activation [29], and of OTR expression in association with CCAAT/enhancer binding protein (C/EBP) activation [30]. Our findings are supported by the work of other groups, who showed that stretch activated mitogen-activated protein kinase (MAPK) in uterine smooth muscle cells both *in vivo* and *in* vitro [31,32]. We also demonstrated that stretch in myometrium contributes to the increase in myometrial IL-8 synthesis associated with the onset of labour [33] and stretch of the amnion may contribute to increased expression of COX-2 and other AP-1 and nuclear factor-kappaB (NF-kappaB) regulated genes with the onset of labour [34].

The changes seen in T-PTL myometrium were similar to those induced by stretch of myometrial cells *in vitro*, but there were inconsistent changes in mRNA and proteins levels. This may reflect the delay in between mRNA and protein changes, miRNA expression, differences in metabolism and the fact that although stretch may have initiated the process, labour itself will have exerted changes in gene expression. The combination of *in vitro* stretch and inflammation may be closer to the *in vivo* situation in pregnancy, since inflammation is common to most labours and all uteri are under some degree of stretch. Generally, although not directly compared, with either stimulus alone, the combination was noted to have more marked effects [35].

Overall, these data suggest that a decline in PKA expression in multiple pregnancies may lead to an increase in OTR expression and promotes the onset of labour. Although there are likely to be several systems involved in the control of myometrial contractility, these data support our earlier publication, which demonstrated a key role for the cAMP effector system in determining the timing of the onset of labour. The similarity in the changes in early labour in term singleton and T-PTL raises the possibility that uterine stretch is a common factor that drives the changes in the cAMP effector system and the onset of labour.

## Supporting information

S1 Fig The levels of adenylyl cyclase (AC2,3&9) in preterm myometrium. Human myometrial tissue samples were snap frozen at -80°C for mRNA and protein extraction and cAMP analysis. The levels of AC2, AC3, and AC9 mRNA **(A, C, E)** and protein **(B, D, F)** were measured using quantitative rt-PCR and western blotting respectively. Blots were probed with AC2, AC3, and AC9 antibody, and GAPDH was used as a loading control. These are the n values for protein samples; AC2, AC3: PTNL (chorio) n = 8, CA-PTL n = 8, PTNL (idio) n = 8, I-PTL n = 4, T-PTNL n = 8, T-PTL n = 6; AC9: PTNL (chorio) n = 8, CA-PTL n = 8, PTNL (idio) n = 7, I-PTL n = 4, T-PTNL n = 8, T-PTL n = 6. *P<0.05, **P<0.01, ***P<0.001 (n = 4–8 in each group).
(PPTX)

S2 Fig The levels of cAMP in preterm myometrium. Human myometrial tissue samples were snap frozen at -80°C cAMP analysis. The cAMP assay was subsequently performed using the cAMP Chemiluminescent Immunoassay kit (Arbor Assays, USA) following the manufacturer’s instructions. These are the n values for the levels of cAMP; PTNL (chorio) n = 19, CA-PTL n = 14, I-PTL n = 7, T-PTNL n = 13, T-PTL n = 6. *P<0.05, **P<0.01, ***P<0.001 (n = 6–19 in each group).
(PPTX)

S3 Fig The levels of cAMP-response-element-binding-protein-binding protein (CBP), inducible cAMP early repressor (ICER) and cAMP response element binding protein (CREB), phospho CREB in preterm myometrium. Human myometrial tissue samples were snap frozen at -80°C for mRNA and protein extraction. The levels of CBP ICER and CREB mRNA (A, C, E) and protein (B, D, F, G) were measured using quantitative rt-PCR and western blotting respectively. Blots were probed with ICER, CBP, CREB, and phospho CREB antibody, and GAPDH was used as a loading control. These are the n values for protein samples; CBP: PTNL (chorio) n = 17, CA-PTL n = 9, PTNL (idio) n = 17, I-PTL n = 11, T-PTNL n = 9, T-PTL n = 8; ICER and phosphor CREB; PTNL (chorio) n = 17, CA-PTL n = 11, PTNL (idio) n = 17, I-PTL n = 11, T-PTNL n = 9, T-PTL n = 8. *P<0.05, **P<0.01, ***P<0.001 (n = 8–17 in each group).
(PPTX)

S1 Table Demographic data for preterm myometrial tissue samples collected. (DOCX)

S2 Table Primary antibodies. (DOCX)

S3 Table Primer pair sequences with gene accession numbers. (DOCX)

S1 Raw images (PDF)
