# Supplementary material for: Changes in cAMP effector predominance are associated with increased oxytocin receptor expression in twin but not infection-associated or idiopathic preterm labour
Source: PLoS One. 2020 Nov 30;15(11):e0240325. doi: 10.1371/journal.pone.0240325 (PMC7703985; doi:10.1371/journal.pone.0240325)
Supplement: S1 Fig — Human myometrial tissue samples were snap frozen at -80°C for mRNA and protein extraction and cAMP analysis. The levels of AC2, AC3, and AC9 mRNA (A, C, E) and protein (B, D, F) were measured using quantitative rt-PCR and western blotting respectively. Blots were probed with AC2, AC3, and AC9 antibody, and GAPDH was used as a loading control. These are the n values for protein samples; AC2, AC3: PTNL (chorio) n = 8, CA-PTL n = 8, PTNL (idio) n = 8, I-PTL n = 4, T-PTNL n = 8, T-PTL n = 6; AC9: PTNL (chorio) n = 8, CA-PTL n = 8, PTNL (idio) n = 7, I-PTL n = 4, T-PTNL n = 8, T-PTL n = 6. *P<0.05, **P<0.01, ***P<0.001 (n = 4–8 in each group). (PPTX) [file pone.0240325.s001.pptx]

## Slide 1
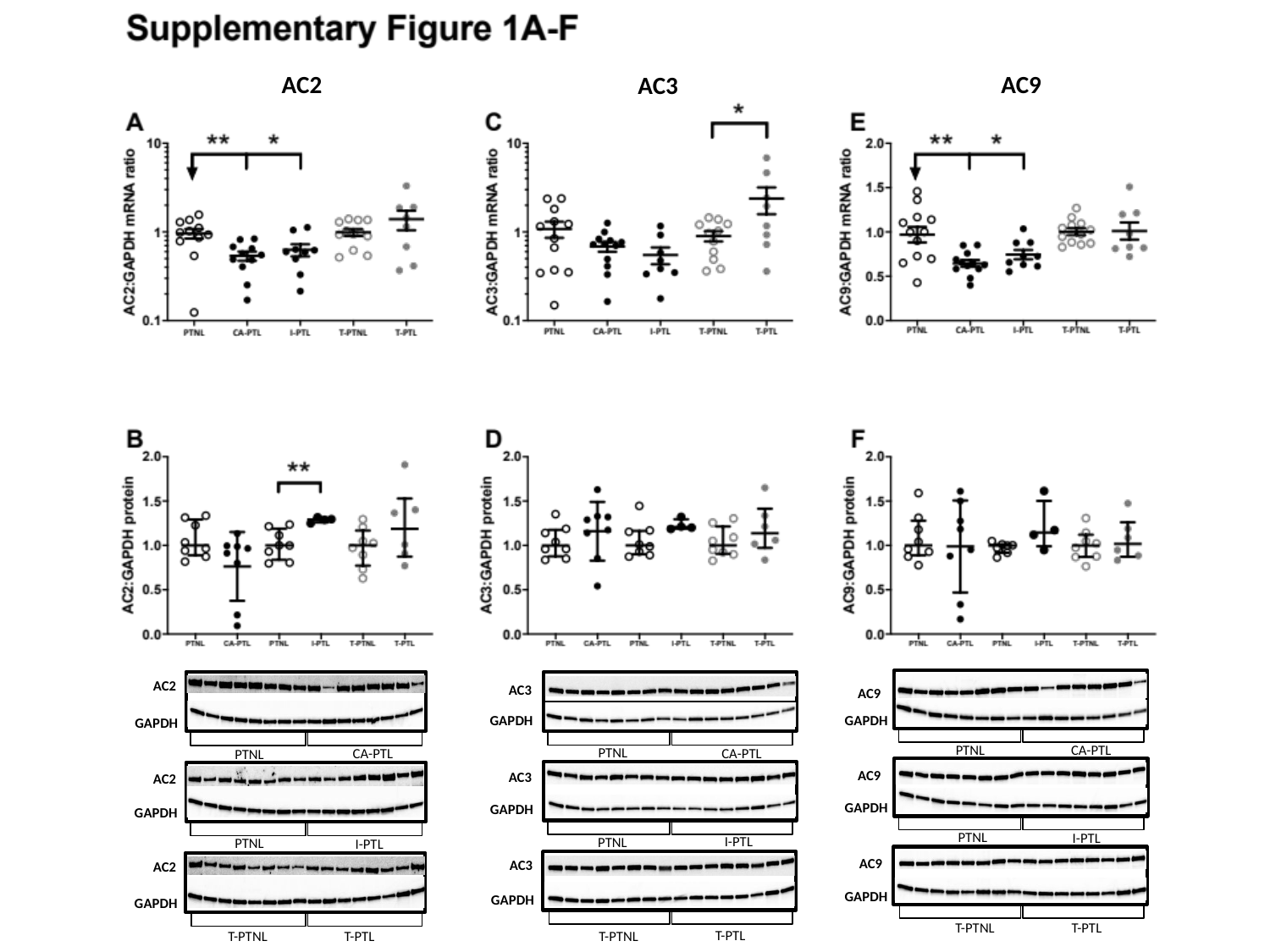

AC9
AC2
AC3
AC2
GAPDH
PTNL
CA-PTL
GAPDH
PTNL
I-PTL
GAPDH
GAPDH
CA-PTL
PTNL
GAPDH
PTNL
I-PTL
GAPDH
GAPDH
PTNL
CA-PTL
GAPDH
I-PTL
PTNL
GAPDH
AC3
AC9
AC9
AC3
AC2
AC9
AC3
AC2
T-PTL
T-PTNL
T-PTL
T-PTL
T-PTNL
T-PTNL
