# Supplementary figures and images for: Changes in cAMP effector predominance are associated with increased oxytocin receptor expression in twin but not infection-associated or idiopathic preterm labour
Source: PLoS One. 2020 Nov 30;15(11):e0240325. doi: 10.1371/journal.pone.0240325 (PMC7703985; doi:10.1371/journal.pone.0240325)

## Slide 1
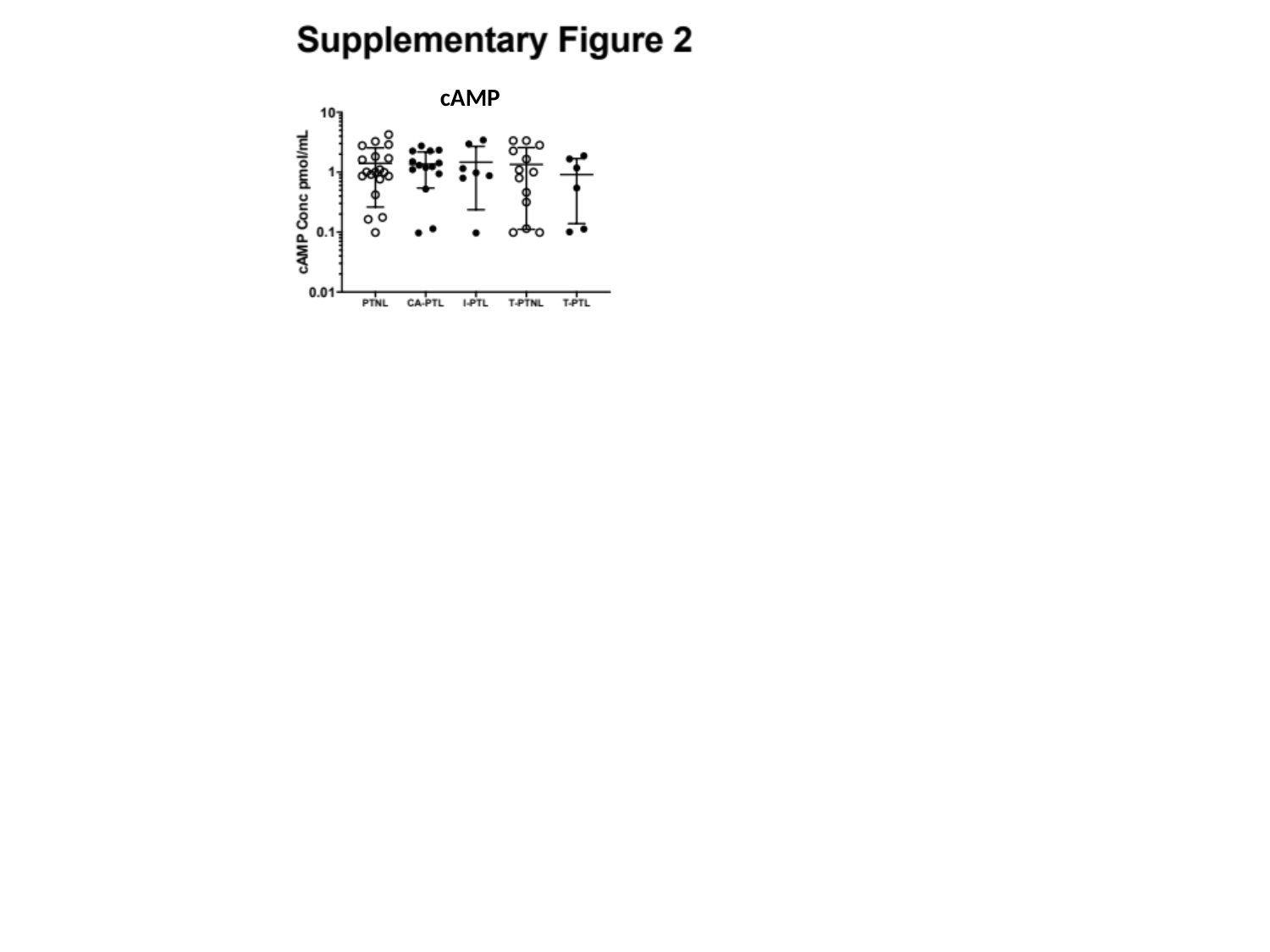

cAMP

Supplement: S2 Fig — Human myometrial tissue samples were snap frozen at -80°C cAMP analysis. The cAMP assay was subsequently performed using the cAMP Chemiluminescent Immunoassay kit (Arbor Assays, USA) following the manufacturer’s instructions. These are the n values for the levels of cAMP; PTNL (chorio) n = 19, CA-PTL n = 14, I-PTL n = 7, T-PTNL n = 13, T-PTL n = 6. *P<0.05, **P<0.01, ***P<0.001 (n = 6–19 in each group). (PPTX) [file pone.0240325.s002.pptx]
