# Supplementary material for: Changes in cAMP effector predominance are associated with increased oxytocin receptor expression in twin but not infection-associated or idiopathic preterm labour
Source: PLoS One. 2020 Nov 30;15(11):e0240325. doi: 10.1371/journal.pone.0240325 (PMC7703985; doi:10.1371/journal.pone.0240325)
Supplement: S3 Fig — Human myometrial tissue samples were snap frozen at -80°C for mRNA and protein extraction. The levels of CBP ICER and CREB mRNA (A, C, E) and protein (B, D, F, G) were measured using quantitative rt-PCR and western blotting respectively. Blots were probed with ICER, CBP, CREB, and phospho CREB antibody, and GAPDH was used as a loading control. These are the n values for protein samples; CBP: PTNL (chorio) n = 17, CA-PTL n = 9, PTNL (idio) n = 17, I-PTL n = 11, T-PTNL n = 9, T-PTL n = 8; ICER and phosphor CREB; PTNL (chorio) n = 17, CA-PTL n = 11, PTNL (idio) n = 17, I-PTL n = 11, T-PTNL n = 9, T-PTL n = 8. *P<0.05, **P<0.01, ***P<0.001 (n = 8–17 in each group). (PPTX) [file pone.0240325.s003.pptx]

## Slide 1
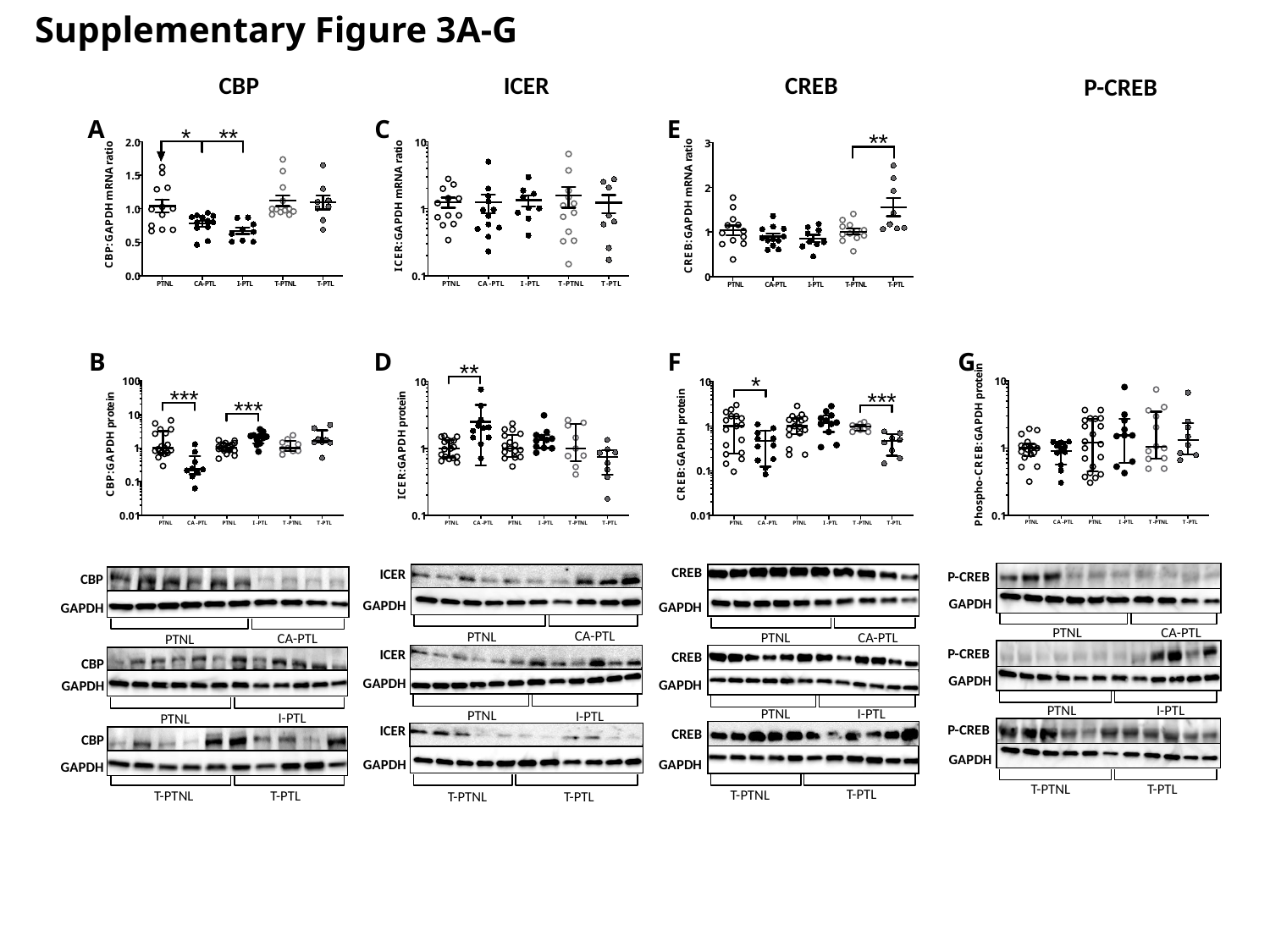

Supplementary Figure 3A-G
A
C
E
*
**
o
2.0
10
i
t
a
r
A
1.5
N
R
m
H
1.0
1
D
P
A
G
:
0.5
P
B
C
0.1
0.0
P
T
N
L
C
A
-
P
T
L
I
-
P
T
L
T
-
P
T
N
L
T
-
P
T
L
PT
N
L
C
A
-PT
L
I
-PT
L
T
-PT
N
L
T
-PT
L
**
o
o
3
i
i
t
t
a
a
r
r
A
A
N
N
R
R
2
m
m
H
H
D
D
P
P
A
A
1
G
G
:
:
B
R
E
E
R
C
I
C
0
P
T
N
L
C
A
-
P
T
L
I
-
P
T
L
T
-
P
T
N
L
T
-
P
T
L
B
D
F
G
**
10
10
n
i
e
t
o
r
p
1
H
D
P
1
A
G
:
0.1
R
E
C
I
0.1
0.01
PT
N
L
C
A
-PT
L
PT
N
L
I
-PT
L
T
-PT
N
L
T
-PT
L
n
i
e
t
10
o
r
p
H
D
P
A
G
:
B
1
E
R
C
-
o
h
p
s
o
h
0.1
P
PT
N
L
C
A
-PT
L
PT
N
L
I
-PT
L
T
-PT
N
L
T
-PT
L
PT
N
L
C
A
-PT
L
PT
N
L
I
-PT
L
T
-PT
N
L
T
-PT
L
*
100
***
n
i
***
e
t
o
10
r
p
H
D
P
1
A
G
:
P
0.1
B
C
0.01
PT
N
L
C
A
-PT
L
PT
N
L
I
-PT
L
T
-PT
N
L
T
-PT
L
n
***
i
e
t
o
r
p
H
D
P
A
G
:
B
E
R
C
CREB
CBP
ICER
P-CREB
CREB
ICER
P-CREB
CBP
GAPDH
CA-PTL
PTNL
GAPDH
PTNL
I-PTL
GAPDH
GAPDH
CA-PTL
PTNL
GAPDH
PTNL
I-PTL
GAPDH
GAPDH
CA-PTL
PTNL
GAPDH
PTNL
I-PTL
GAPDH
GAPDH
CA-PTL
PTNL
GAPDH
I-PTL
PTNL
GAPDH
P-CREB
ICER
CREB
CBP
P-CREB
ICER
CREB
CBP
T-PTL
T-PTNL
T-PTL
T-PTNL
T-PTL
T-PTNL
T-PTL
T-PTNL
