# Supplementary material for: Changes in cAMP effector predominance are associated with increased oxytocin receptor expression in twin but not infection-associated or idiopathic preterm labour
Source: PLoS One. 2020 Nov 30;15(11):e0240325. doi: 10.1371/journal.pone.0240325 (PMC7703985; doi:10.1371/journal.pone.0240325)
Supplement: S1 Table — (DOCX) [file pone.0240325.s004.docx]

Supplementary Table 1. Demographic data for preterm myometrial tissue samples collected.

|  | PTNL | Chorioamnionitis PTL | Idiopathic PTL | Twins NL | Twins PTL |
| --- | --- | --- | --- | --- | --- |
| No of samples | 17 | 12 | 11 | 12 | 8 |
| Mean gestational age (GA±SD) | - - - - 1. 30.2±3.5 | - - - - 1. 29.5±2.9 | - - - - 1. 35.4±1.8 | - - - - 1. 35.2±1.1 | - - - - 1. 34.7±1.36 |
| Maternal characteristics |  |  |  |  |  |
| Age | - - - - 1. 36.2±6.8 | - - - - 1. 33.4±5.3 | - - - - 1. 34.2±5.3 | - - - - 1. 38±5.1 | - - - - 1. 32.8±6.3 |
| BMI | - - - - 1. 26.9±4.2 | - - - - 1. 24.2±4.8 | - - - - 1. 26.0±5.9 | - - - - 1. 22.2±2.9 | - - - - 1. 20.8±2.4 |
