# Supplementary material for: Changes in cAMP effector predominance are associated with increased oxytocin receptor expression in twin but not infection-associated or idiopathic preterm labour
Source: PLoS One. 2020 Nov 30;15(11):e0240325. doi: 10.1371/journal.pone.0240325 (PMC7703985; doi:10.1371/journal.pone.0240325)
Supplement: S2 Table — (DOCX) [file pone.0240325.s005.docx]

**Supplementary Table 2. Primary Antibodies**

**Primary antibodies**

PKAR2α (45-55 kDa): Abcam Ltd., ab38949, Cambridge, UK

EPAC1 (100 kDa): New England BioLabs Ltd., 4155, Herts, UK

GAPDH (38kDa): Millipore MAB374, Watford, UK

OTR-R (C-20) (66 kDa): Santa Cruz Biochemicals, SC-8013, Texas, USA

PKA C-α (42 KDa): New England BioLabs Ltd., 4782, Herts, UK

Gαs (46 kDa): Abcam Ltd., ab83735, Cambridge, UK

ICER (43KDa): Santa Cruz Biochemicals, SC-440, Texas, USA

PDE4B (83 kDa): Abcam Ltd., ab112014, Cambridge, UK

AKAP79 (79kDa): New England BioLabs Ltd., D28G3, Herts, UK

CBP (C-20) (265 kDa): Santa Cruz Biochemicals, SC-583, Texas, USA

CREB (43kDa): Millipore, 04-767, Watford, UK

phospho-CREB (Ser133): New England BioLabs Ltd., 9191, Herts, UK

A cyclase 2 (F7) Santa Cruz Biochemicals, SC-514938, Texas, USA

A cyclase 3 (116 kDa) Thermo fisher, PA1-31191, Massachusetts, USA

A cyclase 9 (161 kDa) Thermo fisher, PA5-36919, Massachusetts, USA
