# Supplementary material for: Changes in cAMP effector predominance are associated with increased oxytocin receptor expression in twin but not infection-associated or idiopathic preterm labour
Source: PLoS One. 2020 Nov 30;15(11):e0240325. doi: 10.1371/journal.pone.0240325 (PMC7703985; doi:10.1371/journal.pone.0240325)
Supplement: S3 Table — (DOCX) [file pone.0240325.s006.docx]

**Supplementary Table 3. Primer pair sequences with gene accession numbers.**

| **Name** | **Primer sequence (5**’**–3**’**)** | **GenBank/**  **EMBL accession no.** |
| --- | --- | --- |
| GAPDH | F: tgatgacatcagaaggtggtgaag  R: tccttggaggccatgtaggccat | BC014085 |
| OTR | F: agaagcactcgcgcctctt  R: aggtgatgtcccacagcaact | NM000916 |
| 11βHSD1 | F: accttcgcagagcaatttgt  R: gccagagaggagacgacaac | NM_005525 |
| MKP-1 | F: cagctgctgcagtttgagtc  R: aggtagctcagcgcactgtt | NM­_004417 |
| PTGES | F:catgtgagtccctgtgatgg  R:ctgcagcaaagacatccaaa | NM_004878 |
| GPR125 | F:ttggcgcagatgtgatagag  R:aagttggctgcttccacagt | NM_145290 |
| CCL8 | F:tcacctgctgctttaacgtg  R:atccctgacccatctctcct | NM_005623 |
| PDE4B | F:ccctgttgtccagtccaact  R:tgcagactctcacggtgaac | NM_001037339 |
| G-alpha-S | F:gaggcttctggcctacactg  R:gcaaggtctgggacctgtaa | NM_000516 |
| GPR124 | F:ctggaagagcgaaactaccg  F:gcgtgtttctgggattgtct | NM_032777 |
| CREB3L1 | F:cagatggctgggaaatcaac  R:tccttggagtgggagaagtc | NM_052854.3 |
| GUCY1A3 | F:caagttgtgcaagccaagaa  R: atccagctctccacactgct | NM_000856 |
| PRKG1 | F:caggcccagatcctatgaaa  R:ccctcaaaccatttgtgctt | NM_001098512 |
| PKAR2α | F:cctagcagatttaatagacg  R:atcatctccttggtcaatga | NM_001144956.1 |
| AKAP79 | F:cggaaagatggtgatgaggt  R:tactggctgctgatggtctg | NM_004857 |
| EPAC1 | F:ccttctgtctccctgactgc  R:ctgcttgacctcctttcagg | NM_001098531 |
| CREB | F:gcgaagggaaattctttcaa  R:cctctctctttcgtgctgct | AY347527.1 |
| CBP | F:cacgtacacacccacacaca  R:gagcgcttgcatgatttaca | [NM_001079846](http://www.ncbi.nlm.nih.gov/entrez/viewer.fcgi?val=NM_001079846) |
| ICER | F: cctccaccaggtgctacaat  R: tttgcgtgttgcttcttctg | [NM_001267562](http://www.ncbi.nlm.nih.gov/entrez/viewer.fcgi?val=NM_001267562) |
| AC2 | F: gccttcatcctcttcgtctg  R: tggagggattgtttcctctg | [NM_020546](http://www.ncbi.nlm.nih.gov/entrez/viewer.fcgi?val=NM_020546) |
| AC3 | F: tgtgctgtggtcttctccag  R: gaagatctgggcggttatga | [NM_004036](http://www.ncbi.nlm.nih.gov/entrez/viewer.fcgi?val=NM_004036) |
| AC9 | F: cctcagtgcagaaccacaga  R: gcaattgcaactcaaagcaa | [NM_001116](http://www.ncbi.nlm.nih.gov/entrez/viewer.fcgi?val=NM_001116) |
