# Supplementary material for: Changes in cAMP effector predominance are associated with increased oxytocin receptor expression in twin but not infection-associated or idiopathic preterm labour
Source: PLoS One. 2020 Nov 30;15(11):e0240325. doi: 10.1371/journal.pone.0240325 (PMC7703985; doi:10.1371/journal.pone.0240325)

Figure 1B PKAc (42 kDa)

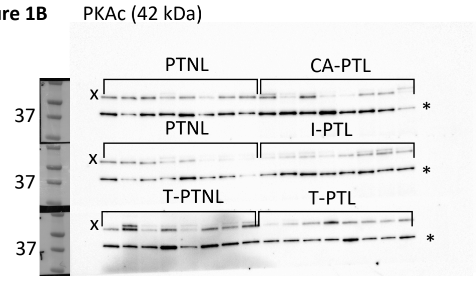

GAPDH for PKAc (36 kDa)

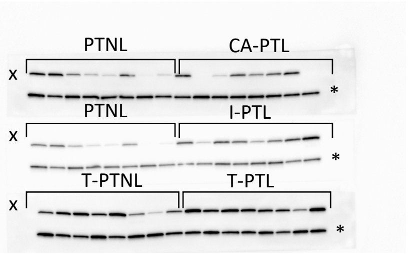

Figure 1D PKAR2α (45-55 kDa)

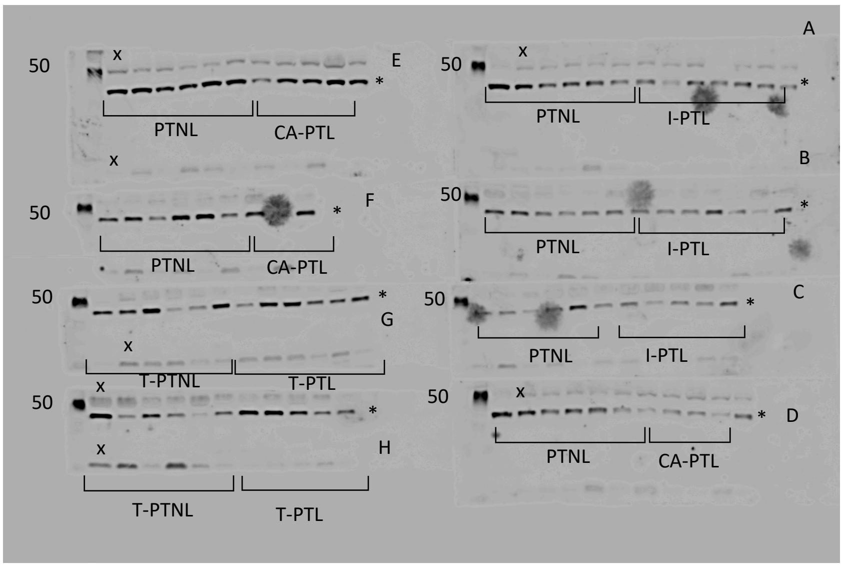

GAPDH (36 kDa) (for PKAR2α - equivalent blots and labelling)

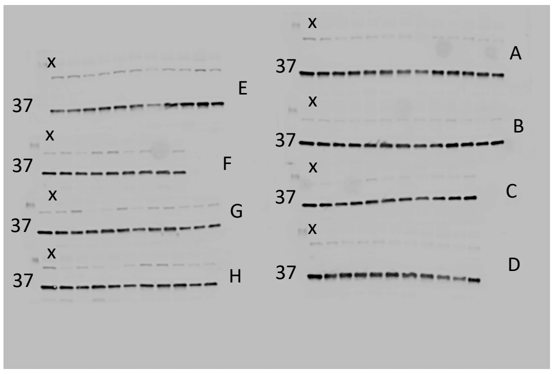

Figure 1F Epac1 (100 kDa)

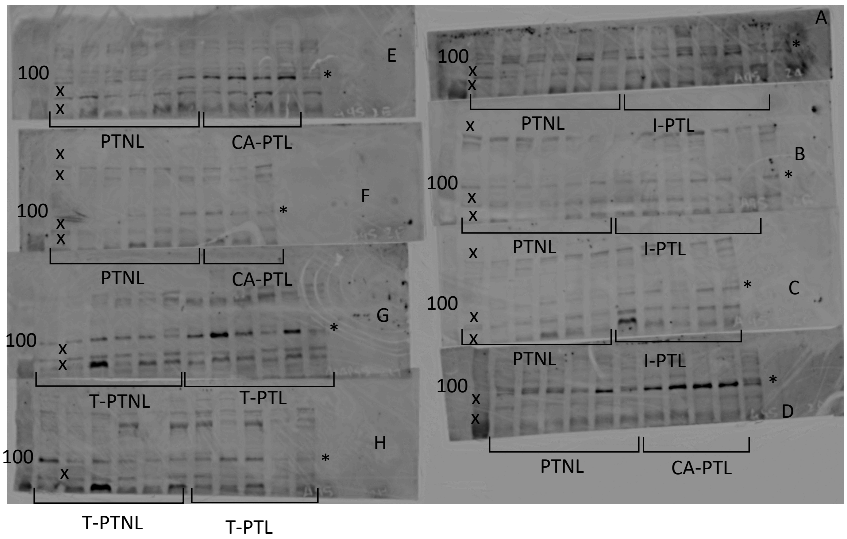

GAPDH (36 kDa) (for Epac1 - equivalent blots and labelling)

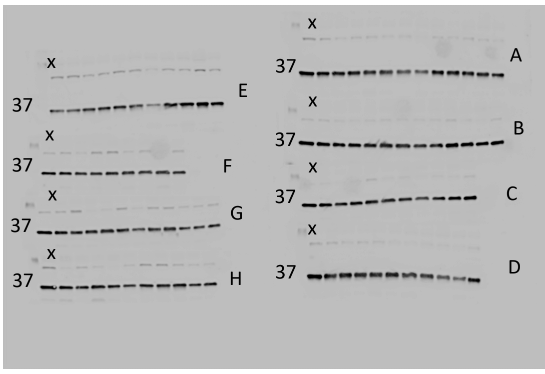

Figure 2B **GαS** (46 kDa)

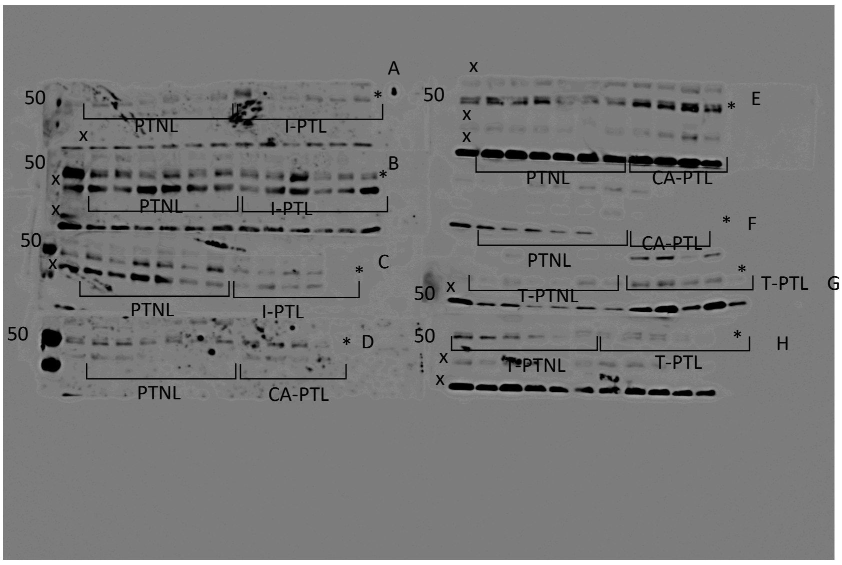

GAPDH (36 kDa) (for GαS - equivalent labelling)

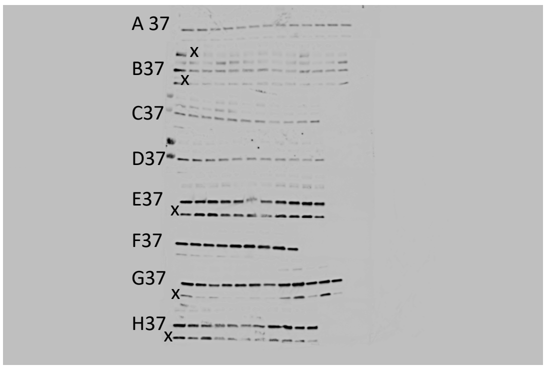

Figure 2D **PDE4B** (83 kDa)

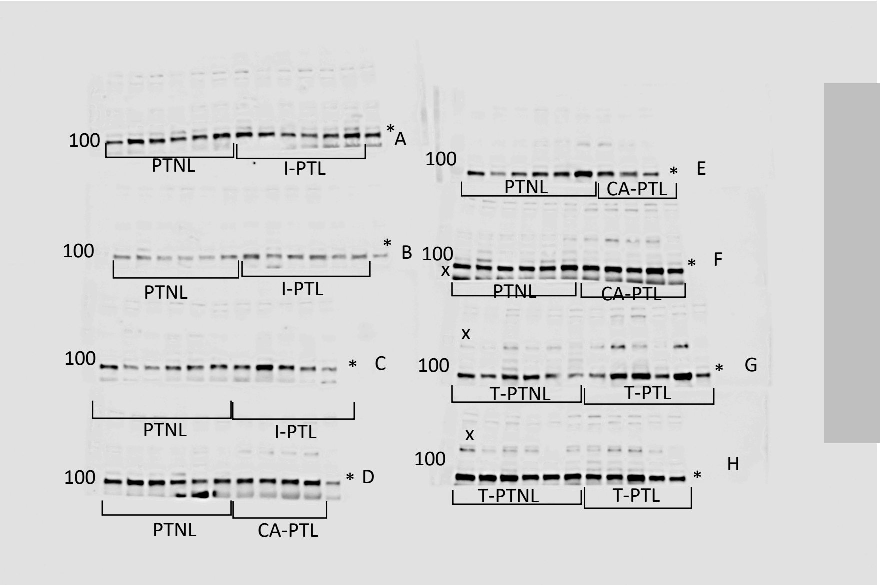

GAPDH (36 kDa) (for PDE4B - equivalent labelling)

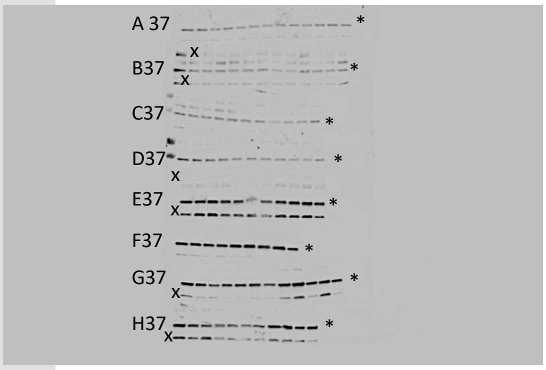

Figure 2F **AKAP79** (79 kDa)

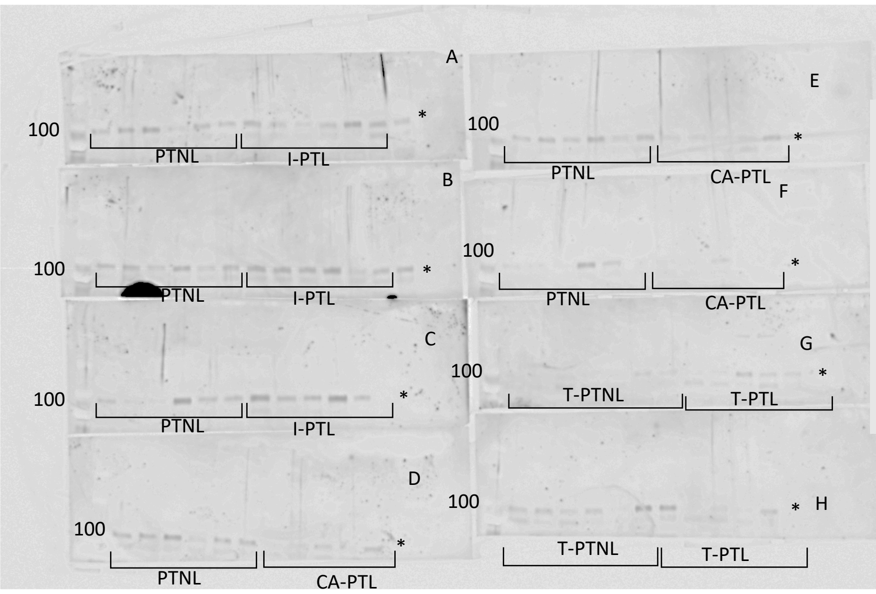

GAPDH (36 kDa) (for AKAP79 - equivalent labelling)

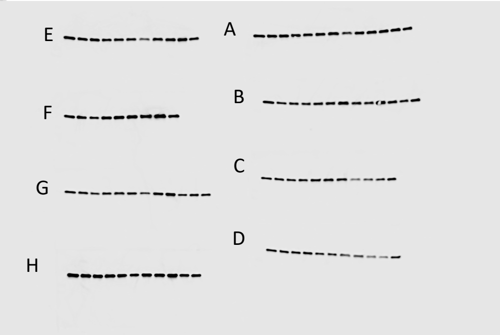

Figure 3B **OTR** (66 kDa)

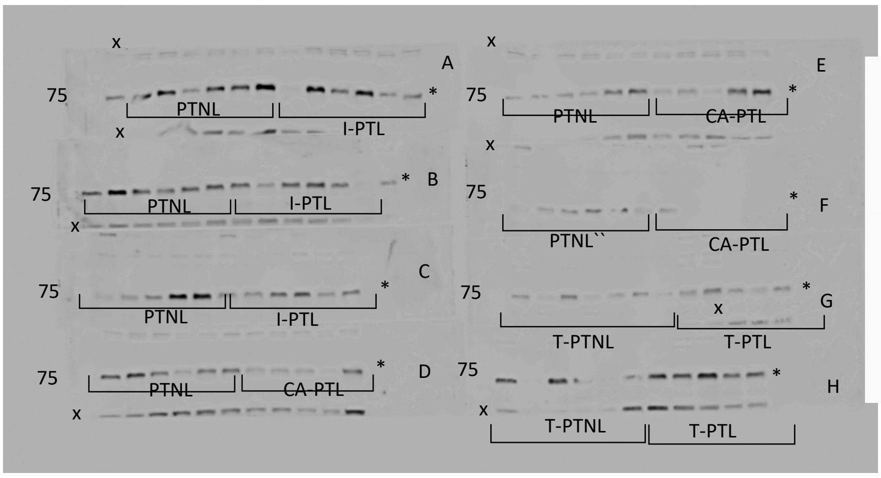

GAPDH (36 kDa) (for OTR - equivalent labelling)

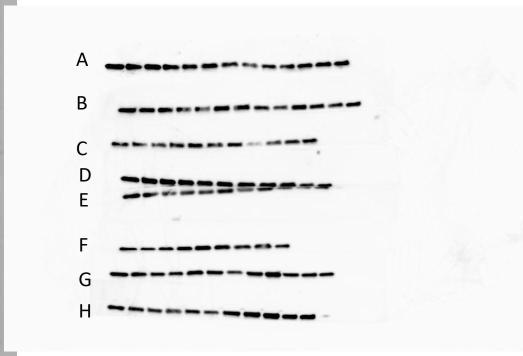

SF 1B **AC2** (124 kDa) GAPDH (36 kDa) (for AC2 - equivalent labelling)

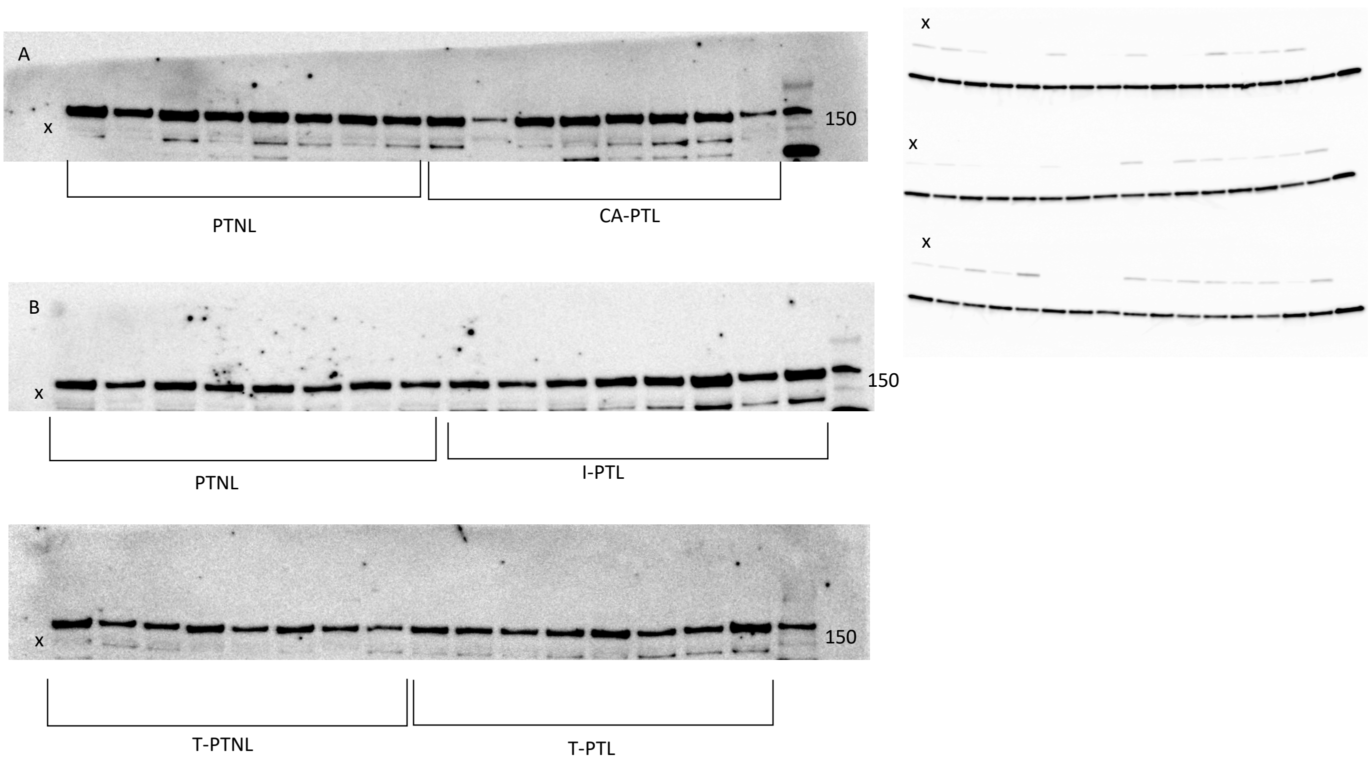

SF 1D **AC3** (116 kDa) GAPDH (36 kDa) (for AC3 - equivalent labelling)

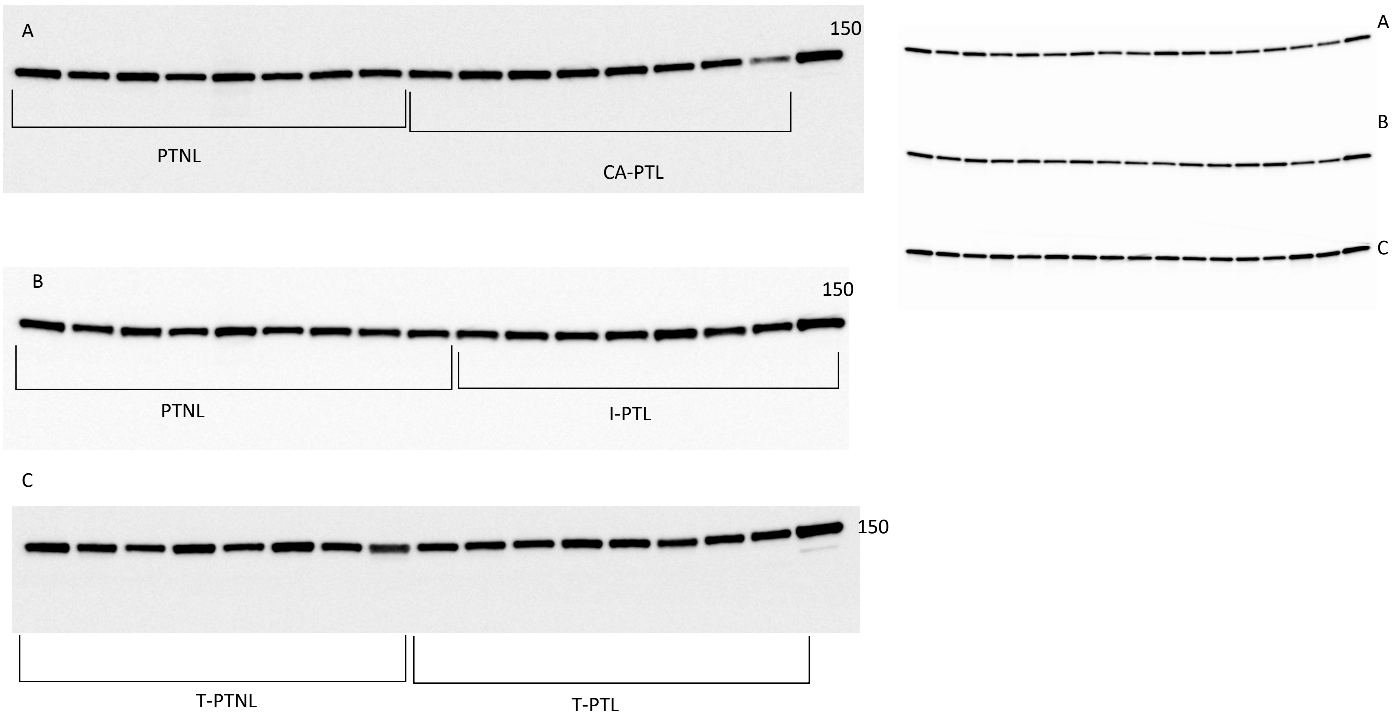

SF 1F **AC9** (161 kDa)

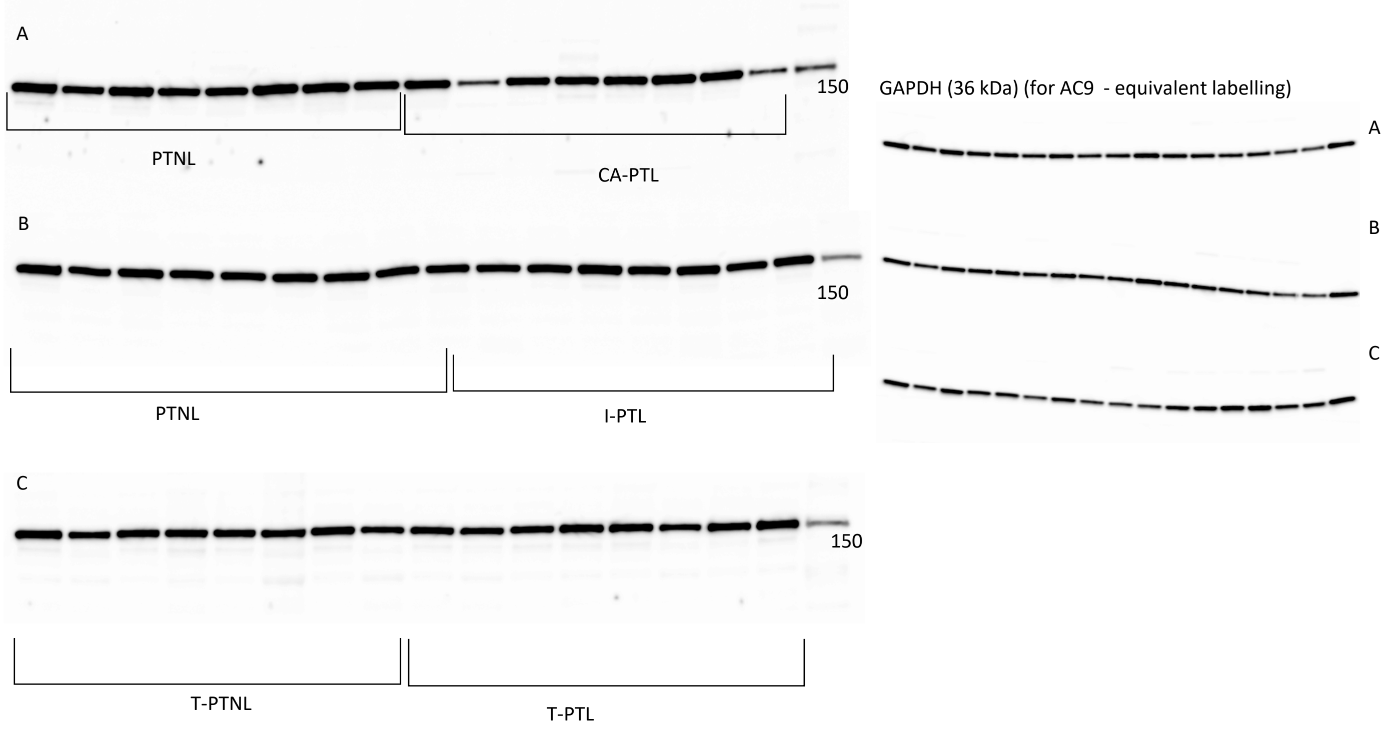

SF 3B **CBP** (265 kDa)

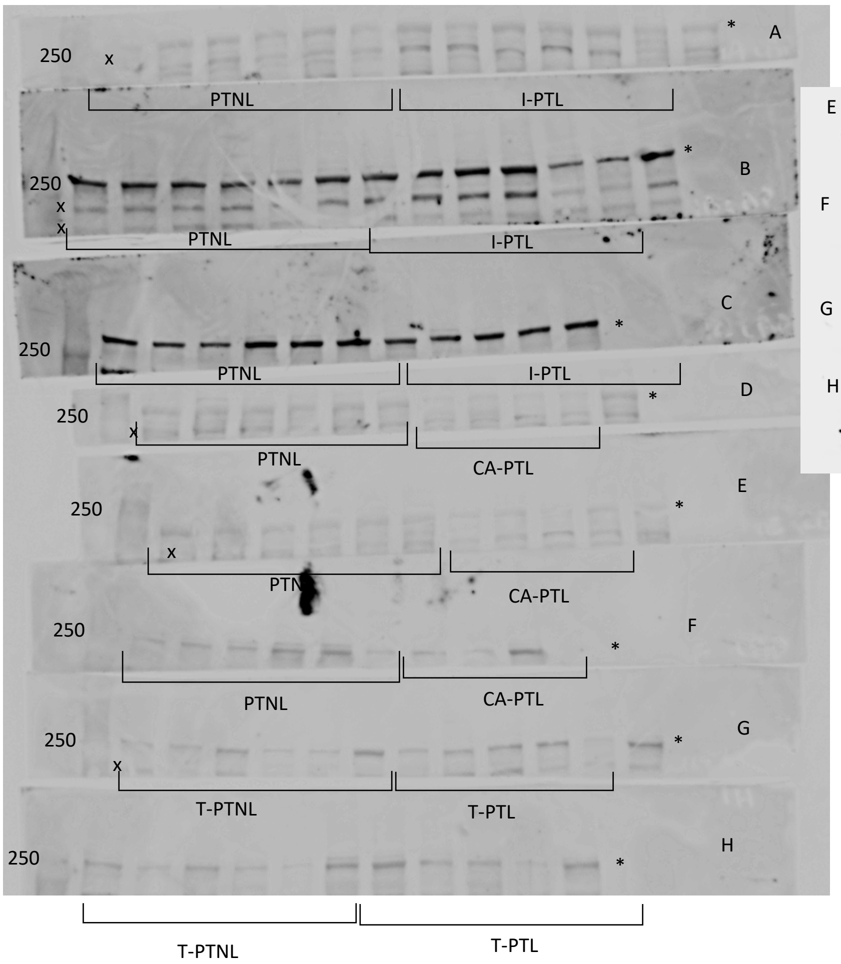

GAPDH (36 kDa) (for CBP - equivalent labelling)

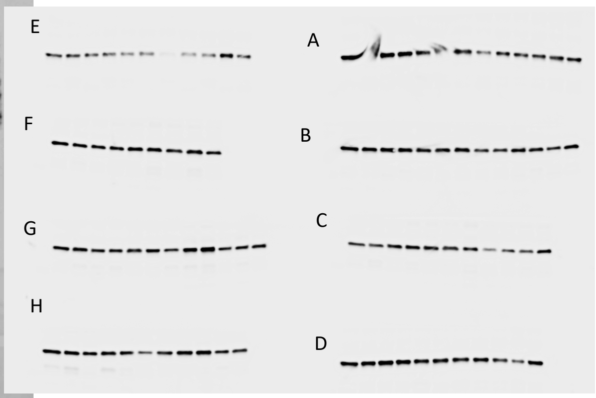

SF 3D **ICER** (43 kDa)

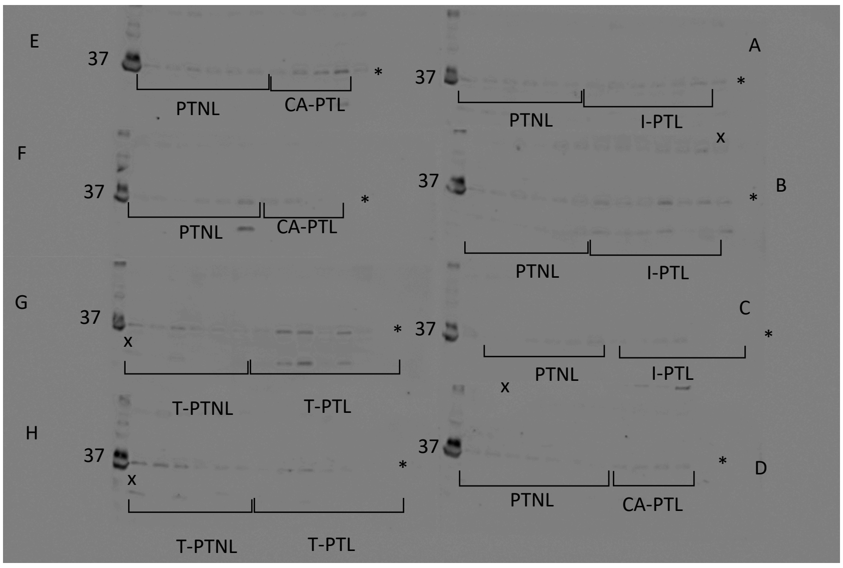

GAPDH (36 kDa) (for ICER - equivalent labelling)

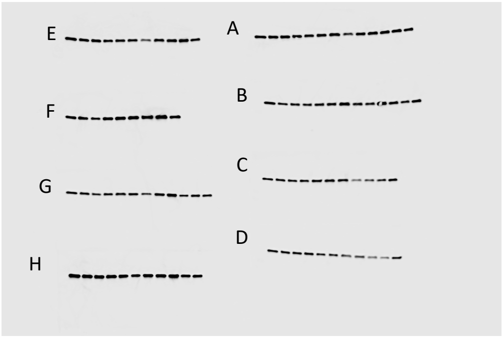

SF 3F **CREB** (43 kDa)

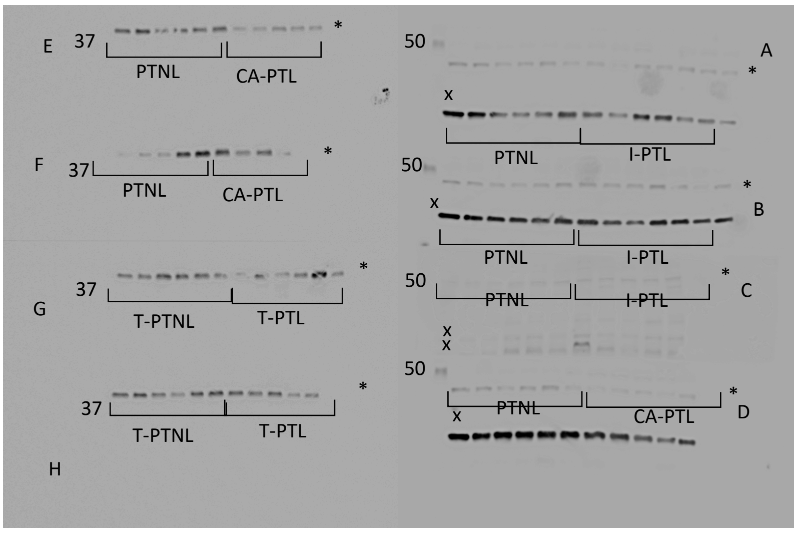

GAPDH (36 kDa) (for CREB - equivalent labelling)

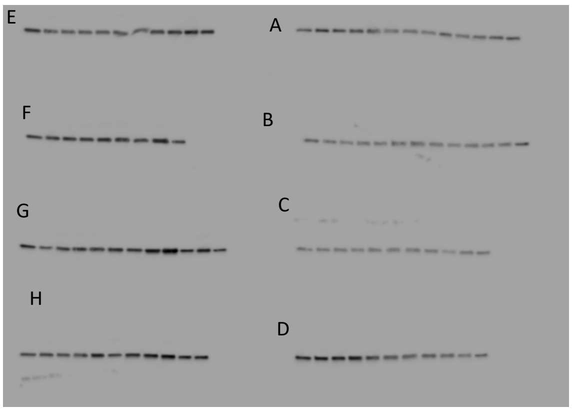

SF 3G **Phospho-CREB** (43 kDa)

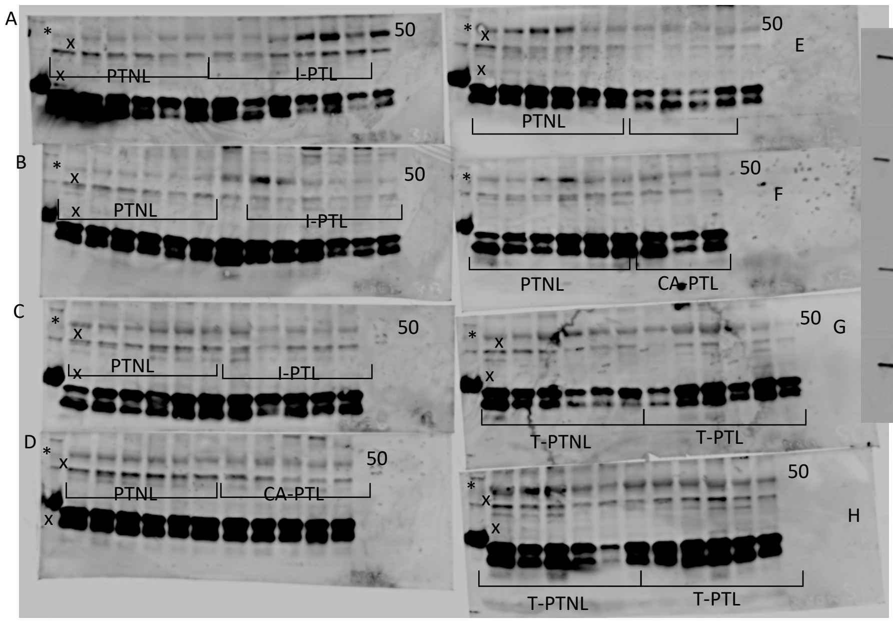

GAPDH (36 kDa) (for Phospho-CREB - equivalent labelling)

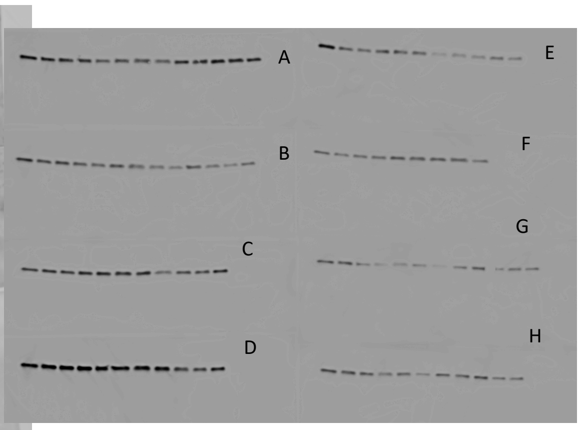

Supplement: S1 Raw images — (PDF) [file pone.0240325.s007.pdf]
